# Interferon-stimulated gene MCL1 inhibits foot-and-mouth disease virus replication by modulating mitochondrial dynamics and autophagy

**DOI:** 10.1128/jvi.00581-25

**Published:** 2025-06-04

**Authors:** Aishwarya Mogulothu, Danielle Hickman, Sarah Attreed, Paul Azzinaro, Monica Rodriguez-Calzada, Meike Dittmann, Teresa de los Santos, Steven Szczepanek, Gisselle N. Medina

**Affiliations:** 1 Department of Pathobiology and Veterinary Science, University of Connecticut 7712, Storrs, Connecticut, USA; 2 Foreign Animal Disease Research Unit, Plum Island Animal Disease Center, Agricultural Research Service, U.S. Department of Agriculture, Greenport, New York, USA; 3 PIADC Research Participation Program, Oak Ridge Institute for Science and Education (ORISE), Oak Ridge, Tennessee, USA; 4 Millipore Sigma 7521 , Indianapolis, Indiana, USA; 5 Department of Microbiology, New York University Grossman School of Medicine, New York, New York, USA; 6 Center of Excellence for Vaccine Research, University of Connecticut 7712, Storrs, Connecticut, USA; 7 United States Department of Agriculture, National Bio and Agro-Defense Facility (NBAF), Agricultural Research Service, Manhattan, Kansas, USA; The Peter Doherty Institute for Infection and Immunity, Melbourne, Australia

**Keywords:** foot-and-mouth disease virus, interferon-stimulated genes, antiviral agents, interferons, mitochondrial metabolism, autophagy

## Abstract

Interferons (IFNs) and the IFN-stimulated genes (ISGs) that they induce are effective in reducing the replication of foot and mouth disease virus (FMDV). The use of a high-throughput ISG screen identified the ISG myeloid cell leukemia 1 (MCL1) as an ISG with an antiviral effect against an FMDV replicon system. In this study, we demonstrated that overexpression of MCL1 inhibits FMDV replication by reducing approximately 4 logs of virus titers in porcine cells. We then explored the regulatory pathways associated with MCL1 to determine the specific antiviral mechanisms against FMDV. Our findings indicated that the antiviral mechanism does not involve apoptosis regulation or alterations in cell cycle phase heterogeneity. Analysis of mitochondrial function, through measurement of mitochondrial oxygen consumption rate, demonstrated that overexpression of MCL1 results in increased mitochondrial respiration and ATP production, whereas FMDV infection reduces both processes. Moreover, MCL1 overexpression resulted in elongated mitochondrial morphology, contrasting with the fragmented and punctate morphology observed during FMDV infection. Importantly, these changes in mitochondrial dynamics were independent of MCL1’s regulation of mitochondrial calcium flux. We also found that MCL1 overexpression suppresses autophagy, which is known to be necessary for FMDV replication. Our data indicate that MCL1 is a potent antiviral ISG against FMDV and highlight the importance of mitochondrial dynamics and autophagy in FMDV replication.

**IMPORTANCE:**

In this study, we have successfully used a high-throughput ISG screening approach to measure the inhibition of FMDV replication using an RNA replicon system for the first time. This screen led to the identification of the potent antiviral effects of a relatively lesser-known ISG called MCL1. Our findings reveal that MCL1 exerts its antiviral functions through the regulation of mitochondrial dynamics and autophagy. Although mitochondrial dynamics are involved in apoptosis, metabolism, redox homeostasis, stress responses, and antiviral signaling, this pathway has not been thoroughly explored in the context of FMDV infection. Further investigation into mitochondrial dynamics may facilitate the development of improved biotherapeutics for FMDV. Additionally, our studies highlight the significance of autophagy, a pathway that is needed by FMDV for replication. Ultimately, a deep understanding of all mechanisms exploited by FMDV may allow for the rational design of novel therapeutics and vaccines to control FMD.

## INTRODUCTION

Foot-and-mouth disease virus (FMDV) is a positive-sense, single-stranded RNA virus belonging to the genus *Apthovirus* and the family *Picornaviridae*. FMDV causes FMD in cloven-hoofed animals, such as swine and cattle. The disease is characterized by fever, lameness/weakness, and vesicle formation of feet, mouth, and teats of animals (1). Due to the highly contagious nature of FMD, it is considered a viral disease with impactful economic repercussions. FMD is estimated to affect 77% of the world’s livestock population in countries that are part of Africa, Eurasia, and some parts of South America (2). Countries that are FMD-free without vaccination, such as the United States, are under constant threat of outbreaks due to lapses in biosecurity. The World Organisation for Animal Health dictates strategies for control of FMDV outbreaks, and the plans that are employed include destruction of all infected and FMD-susceptible contact animals, strict quarantine protocols, disinfection of premises and infected materials, and disposal of all animal products and carcasses. The costs for these types of control strategies and the interruption of trade can cause severe economic impacts in countries with FMD outbreaks. Employment of vaccination strategies with inactivated vaccines can help mitigate disease outbreaks, but these strategies are not implemented in all countries, thus leading to the endemic status of FMD in various regions around the world (3). In addition, due to the requirement that inactivated vaccines be produced under BSL-3 conditions, they are quite costly. Therefore, novel preventative vaccine strategies and biotherapeutics are needed and desired for subduing the spread of FMDV.

One promising biotherapeutic strategy under investigation is the use of interferon (IFN)-based therapeutics. Usually, infection of host cells with most viruses triggers IFN expression, leading to the subsequent induction of IFN-stimulated genes (ISGs) that display a broad range of antiviral functions. However, FMDV employs mechanisms that block IFN functionality through the inhibition of host transcription and translation, as well as blockage of IFN induction pathways and signaling pathways (4). Therefore, to abrogate the evasion of IFN signaling pathways by FMDV, treatment of the host animals with IFN can be used to reduce the replication of the virus. Thus far, all three classes of IFN have been tested against FMDV using various delivery methods, demonstrating efficacy in controlling disease outcomes in natural hosts (4). As IFN treatment induces a broad antiviral landscape with many ISGs, understanding the specific mechanisms of viral inhibition is crucial. This knowledge will enhance our understanding of virus-host interactions and help develop targeted therapies against FMDV. To elucidate which specific ISGs are inhibitory against FMDV, an ISG screen developed by Schoggins et al. was used (5). This screen used a high-throughput microscopy approach with RFP-tagged lentiviruses to overexpress the ISGs *in vitro* and a FMDV A24-GFP replicon to determine which ISGs were inhibitory to FMDV replication. The results of this screen showed that the ISG MCL1 was among the top candidates for inhibiting FMDV replicon.

MCL1 is an ISG that has various functions in the cell to maintain homeostasis. These functions include apoptosis, cell cycle regulation, autophagy/mitophagy, and mitochondrial morphodynamics (6). Among these, MCL1’s involvement in apoptosis is the most extensively studied. As part of the BCL2 family of proteins that regulate apoptosis, MCL1 exhibits anti-apoptotic functions. During apoptosis, the mitochondrial outer membrane becomes permeabilized, leading to the oligomerization of the pro-apoptotic proteins BAX and BAK on the outer mitochondrial membrane. This process facilitates cytochrome c efflux, which triggers downstream caspase activation. MCL1 counteracts this process by inhibiting the oligomerization of BAX and BAK, thereby preventing apoptosis (7). Numerous studies have identified various FMDV proteins, including VP1, VP3, 2C, 2B, and 3C, as contributors to apoptosis induction (8–12). Therefore, MCL1’s inhibitory role could be linked to its influence on apoptotic pathways.

MCL1 has also been shown to play various roles in cell cycle regulation occurring at all phases (G1, S, G2/M), and this regulation is dependent on MCL1 expression levels as well (6). MCL1 is known to negatively regulate a G1/S checkpoint protein called p18. MCL1 targets p18 for proteolytic degradation, allowing for cell cycle entry and the G1/S transition (13). MCL1 also regulates progression through S phase by interactions with PCNA (14). A proteolytically cleaved isoform of MCL1, called snMCL1, can also inhibit the interaction between CDK1 and its partner cyclin B. This leads to decreased proliferation and blocking of the completion of the mitotic phase (15). Only one study has investigated the effect of FMDV on the cell cycle. The investigators in this study demonstrated that during the G2/M phase, FMDV-infected cells display a higher amount of viral RNA and increased expression of the 3D polymerase. In addition, when the cell cycle phase is arrested at G2/M, more FMDV viral progeny is produced (16). If MCL1 arrests the cell cycle at cell cycle phases other than G2/M, it could explain its role in inhibiting FMDV replication.

Another avenue to be explored is MCL1’s manipulation of mitochondrial dynamics. Mitochondria are important organelles involved in apoptosis, metabolism, redox homeostasis, stress responses, and antiviral signaling. Antiviral signaling occurs through mitochondrial antiviral signaling (MAVS), to mediate innate immunity with IFN and ISGs (17). Studies have shown that mitochondrial morphology affects mitochondrial function. Mitochondria are in a flux between two processes, fission and fusion-transitioning mitochondrial morphology between elongated and tubular to fragmented states. Balance between fission and fusion is essential for maintaining mitochondrial function in the face of various stressors, as it allows for the removal of damaged mitochondrial components while maintaining ATP production (18). One study found that MCL1 deletion leads to loss of tubular mitochondrial structures and reduced ATP production. The authors noted that MCL1 translocates to the inner mitochondrial matrix, where it stabilizes the protein OPA1 to allow for mitochondrial fusion (19). Another study indicated that the splicing variant MCL1S also causes an increase in mitochondrial calcium, leading to hyperfusion and elongation of mitochondria (20). However, there is limited research done on the influence of FMDV on mitochondrial dynamics. One study demonstrated that FMDV influenced mitochondrial function by decreasing mitochondrial membrane potential and mitochondrial pore-opening over time while also increasing mitochondrial calcium and ROS levels (21). FMDV VP3 protein has also been shown to inhibit MAVS protein activation on the mitochondria, leading to the suppression of the IFN response (22). These findings indicate that FMDV manipulates mitochondrial function in a multitude of ways, highlighting the need to investigate its effects on mitochondrial morphodynamics. MCL1 may abrogate the effects FMDV has on mitochondrial function, potentially explaining its role in inhibiting viral replication.

MCL1 has both inhibitory and facilitating roles in autophagy, with its effects depending on tissue type and whether selective or non-selective autophagy is triggered. In most cases, MCL1 inhibits nonselective autophagy, usually triggered by stressors like nutrient deprivation. MCL1 inhibits this type of autophagy through interactions with Beclin-1 (23). However, reports on the role of MCL1 in selective autophagy are conflicting, likely due to tissue-specific differences. In some cases, MCL1 inhibits selective autophagy of mitochondria (mitophagy) in Beclin-1-dependent and -independent pathways (24, 25). However, other studies have shown that MCL1 induces mitophagy to clear away damaged mitochondria (23). In the context of FMDV, autophagy is induced upon entry into the cell to facilitate viral replication (26). In fact, two separate studies have shown that inhibition of autophagy leads to inhibition of FMDV replication (26, 27). In addition to those functions, the induction of autophagy also allows the downregulation of innate immune signaling, as shown in various reports involving FMDV replication and expression of FMDV viral proteins (28–31). Therefore, inhibition of autophagy by MCL1 may contribute to the inhibition of FMDV replication.

In this study, we first validate the inhibitory role of MCL1 in the context of wild-type FMDV infection. We then explore the different regulatory roles of MCL1 to identify which specific role contributes to its antiviral function against FMDV. Our results indicate that MCL1 enhances mitochondrial respiration and the formation of elongated mitochondria, whereas FMDV infection causes decreases in mitochondrial function and induces mitochondrial fragmentation. Furthermore, here we also demonstrate that MCL1 inhibits the induction of autophagy, a pathway required for maximum FMDV replication and spread in host cells.

## RESULTS

### Validation of ISG screen results by overexpression of MCL1 in porcine cells

The discovery of MCL1 as an ISG that inhibits FMDV replication was achieved through the use of a modified high-throughput microscopy ISG screen (5). Baby hamster cells (BHK-21) were seeded in 96-well plates with each well transduced by a different lentivirus expressing human ISG along with RFP, totaling 370 ISGs screened. At 48 h post-transduction, the cells were transfected with an FMDV A24-GFP RNA replicon, which contained all but the capsid viral RNA genomic sequences. Following incubation, the plates were then imaged and analyzed for the total number of GFP, RFP, and double-positive cells. The screen was conducted twice, and the results indicated that out of the 370 ISG, the top five hits were RIG-I, IRF7, MCL1, IFI30, and CXCL10 (Table S1). We therefore decided to concentrate on MCL1, given the previously described role of the other genes in controlling FMDV replication (Table S1).

**Fig 1:**
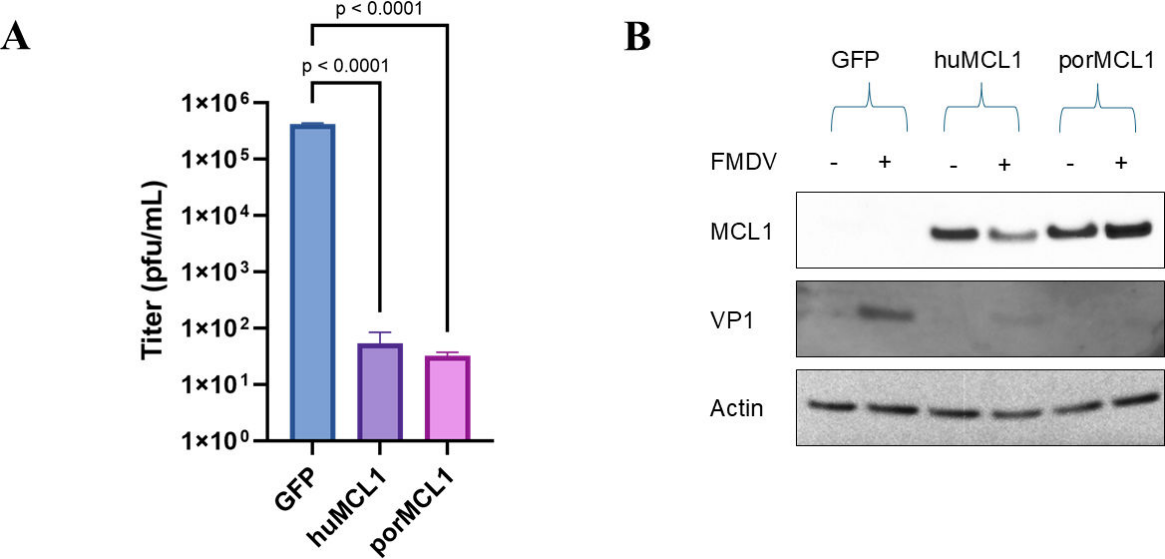
FMDV replication in MCL1-overexpressing porcine cells. (**A**) MGPK-αvβ6 cells were transduced with SCRPSY lentivirus either expressing GFP, huMCL1, or porMCL1. They were then infected with FMDV A12 WT at an MOI of 5 and followed by a 6 h incubation period (*n* = 3). Virus titers were determined by plaque assay on BHK-21 cells. (**B**) Western blot was conducted on lysates collected at 6 h post-infection and probed with antibodies against MCL1, FMDV VP1, and actin. Statistical analysis was performed using one-way ANOVA with the Tukey *post hoc* test.

To validate the results obtained with MCL1 using the replicon system in BHK-21 cells, the overexpression of MCL1 was done in the context of a wild-type (WT) infection in a cell line that is physiologically more relevant. MGPK-αvβ6 cells were transduced with either the SCRPSY lentivirus-expressing GFP (control) or MCL1, and overexpression was confirmed using western blot (Fig. 1B). When comparing the titers of the infected transduced cells, human MCL1 (huMCL1) caused a 4-log decrease in viral titers compared with the GFP cells at 6 h post-infection (Fig. 1A). The antiviral qualities of porcine MCL1 (porMCL1) were also tested, as there is a ~ 10% difference in the amino acid sequences when aligning huMCL1 and porMCL1, and it also demonstrated a similar level of viral titer reduction (Fig. 1A). Henceforth, the rest of the experiments described were done using porMCL1, given its relevance to porcine cells. Knockdown experiments were also done in MGPK-αvβ6 cells using a combination of two siRNAs targeting porcine MCL1. Using western blot analysis, we observed ~70% knockdown of MCL1 protein expression (Fig. S1A and B). However, no statistically significant change in viral titer was observed when compared with the negative control siRNA (Fig. S1C). Therefore, the results of the screen were validated with overexpression of MCL1 in porcine cells. These data indicate that MCL1 has an antiviral effect against FMDV, and the rest of this investigation will focus on the mechanisms underlying this inhibition.

### Inhibition of apoptosis does not affect WT FMDV replication

**Fig 2:**
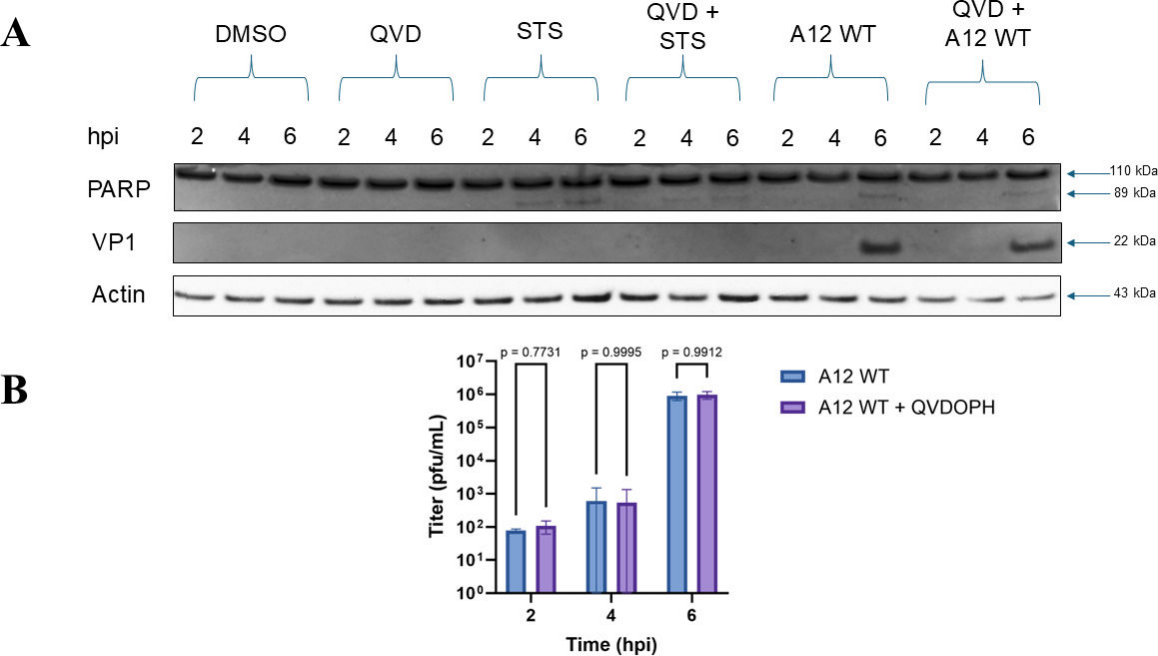
FMDV replication in porcine cells treated with Q-VD-OPH. (**A**) MGPK-αvβ6 cells were treated with DMSO, 1 µM of staurosporine (STS), 20 µM of Q-VD-OPH (QVD) and STS, FMDV A12 WT MOI: 5, or QVD and A12 WT. Lysates were collected at indicated time points and run on a western blot to be probed with antibodies against PARP, actin, or FMDV VP1. (**B**) MGPK-αvβ6 cells were treated with either DMSO or 20 µM of QVD, followed by infection with FMDV A12 WT at an MOI: 5 (*n* = 3). Virus titers were determined by plaque assay on BHK-21 cells. Statistical analysis was performed using 2-way ANOVA with the Tukey post hoc test.

As the most well-characterized function of MCL1 is its role in apoptosis, we first explored this pathway to determine whether this function is relevant to the antiviral activity of MCL1. This was done by simulating the anti-apoptotic function of MCL1 with the pan-caspase inhibitor Q-VD-OPH (QVD). We utilized PARP cleavage as an indicator for apoptosis in our analysis, as seen in Fig. 2A when cells are treated with the apoptosis inducer staurosporine (STS). The STS-induced PARP cleavage was ablated when treated with 20 µM of QVD, demonstrating that caspase function was inhibited (Fig. 2A). However, at all time points, there were no statistically significant differences in titers detected when comparing cells treated with or without QVD (Fig. 2B). An experiment utilizing a higher concentration of QVD (50 µM) also failed to show any differences in viral titer (data not shown). These results indicate that blocking apoptosis by caspase inhibition did not have an effect on FMDV replication. We concluded that the antiviral function of overexpressed MCL1 is unlikely to be due to its role in blocking apoptosis, as the same effect could not be reproduced with a peptide inhibitor of apoptosis.

### MCL1 does not change cell cycle heterogeneity in MGPK αvβ6 cells

**Fig 3:**
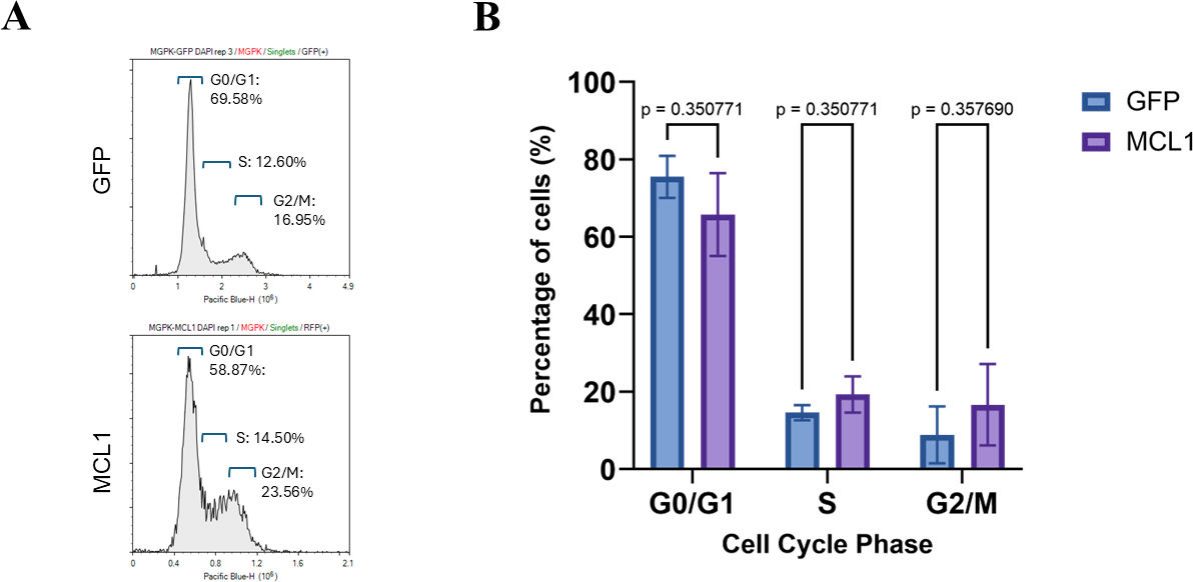
Cell cycle analysis of MCL1 overexpressing porcine cells. MGPK-αvβ6 cells overexpressing either GFP or porMCL1 were collected, fixed, and then stained with DAPI before being analyzed by flow cytometry. (**A**) Representation of the cell counts in each phase of the cell cycle (G0/G1, S, G2/M) based on DAPI fluorescence for both overexpressing cell lines. (**B**) Percentage (%) distribution of each cell cycle phase in MGPK-GFP and MGPK-porMCL1 cells. Statistical analysis was performed using Student’s *t*-test.

The next pathway we explored was the effect of MCL1 on the cell cycle distribution of MGPK-αvβ6 cells, given MCL1’s known regulatory roles in cell cycle checkpoints. MGPK-GFP or porMCL1 cells were analyzed for DNA content using flow cytometry (Fig. 3A). Cells were gated based on GFP or RFP fluorescence to include only transduced cells in the analysis. Although variations were observed in the number of cells at each stage of the cell cycle (G0/G1, S, G2/M) when comparing MGPK-GFP and MGPK-porMCL1 transduced cells, there were no statistically significant differences in the distribution of cells across the various phases of the cell cycle. This means that MCL1 overexpression did not cause an arrest in the cell cycle phase that could be detrimental to FMDV replication. Similarly, when we performed the same assay on MGPK-αvβ6 cells that were either infected or mock-infected, there was also no statistically significant difference in cell cycle distribution (Fig. S2). Therefore, the antiviral function of MCL1 cannot be ascribed to perturbations in the cell cycle, as no statistically significant changes in the distribution of cells in each phase of the cell cycle were observed.

### MCL1 influences mitochondrial function to increase oxidative phosphorylation-related basal respiration and ATP production

**Fig 4:**
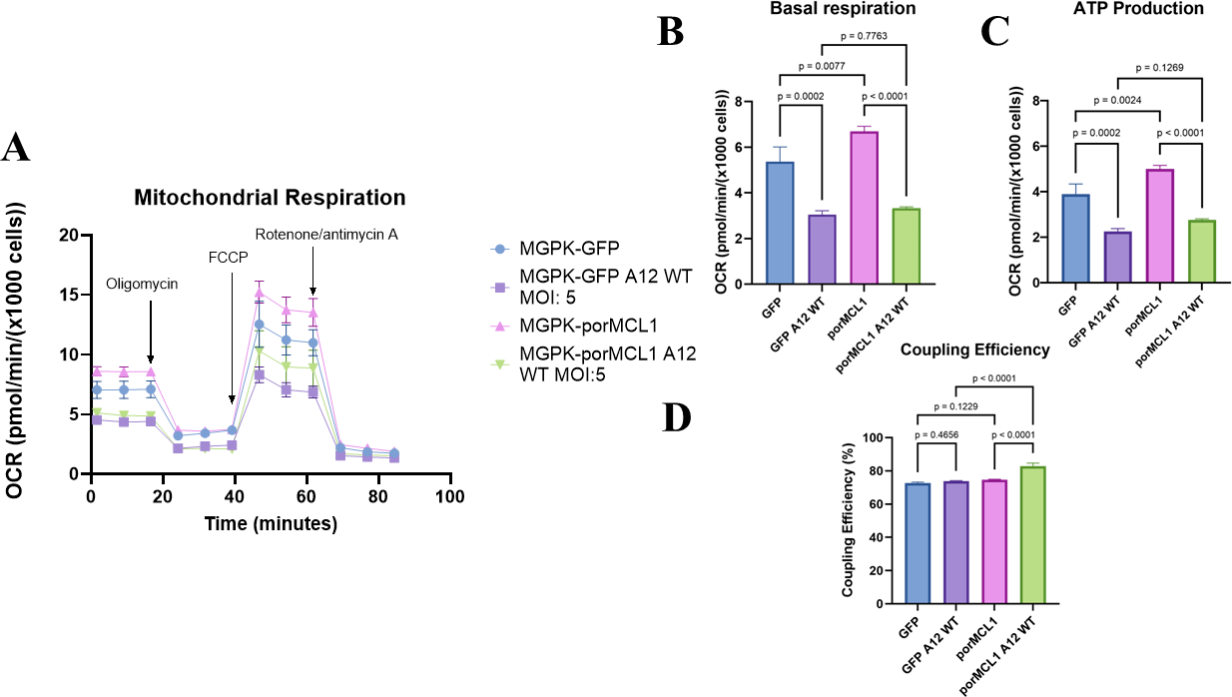
Mito stress test on porcine cells overexpressing MCL1. (**A**) Mito stress test was conducted with a Seahorse bioanalyzer on MGPK-GFP and MGPK-porMCL1 cells that were mock-infected or infected with FMDV A12 WT MOI: 5. Subsequent addition of oligomycin, FCCP, and Rotenone/antimycin A allowed for calculation of different types of respiration occurring in mitochondria. (**B**) Basal respiration-related OCR from mito stress test in (**A**). Basal respiration is oxygen consumption under basal conditions to meet the ATP demand of the cells, and also due to proton leak. (**C**) ATP production OCR from mito stress test in (**A**). ATP production is oxygen consumption required to create ATP and is measured after the addition of oligomycin. (D) Coupling efficiency from mito stress test from (**A**). Coupling efficiency refers to how efficiently mitochondria are at consuming oxygen, coupled to ATP production versus proton leak. It is calculated by dividing ATP-linked OCR by basal respiration-linked OCR. Statistical analysis was performed using one-way ANOVA with the Tukey *post hoc* test.

We next explored MCL1’s role in mitochondrial function. The mitochondrial function of MCL1 was measured with the Agilent Seahorse Mito Stress test, which monitors extracellular flux of oxygen by measuring oxygen consumption rate (OCR). Figure 4A demonstrates the mitochondrial respiratory profile of MGPK-GFP and MGPK-porMCL1 cells that were either mock-infected or FMDV-infected. Statistically significant differences were observed in basal respiration and ATP production between the groups, and not surprisingly, these two processes are related to each other (Fig. 4B and C). Basal respiration reflects the amount of mitochondrial respiration needed to keep up with the ATP demands of the cell. When cells are infected with FMDV, there is a decrease in OCR related to these two parameters (basal respiration and ATP production), which is indicative of mitochondrial dysfunction and fragmentation of mitochondria (18). Interestingly, porMCL1 overexpression resulted in an increase in OCR regarding basal respiration and ATP production. Generally, mitochondria that are elongated have a higher level of basal respiration and generate more ATP (32).

Coupling efficiency refers to the amount of oxygen consumed that is used to drive ATP production (generation of energy used for the cell) versus the amount of oxygen used to drive proton leak (generation of heat). Although there were no differences in the MGPK-GFP cells or in the mock-infected porMCL1 cells, there was a statistically significant increase in coupling efficiency in infected porMCL1 cells (Fig. 4D). This indicates that during an infection, porMCL1 allows for the mitochondria to more efficiently generate ATP with the oxygen consumed instead of being coupled with proton leak. Taken together, the results from the mito stress test indicate that FMDV infection causes mitochondrial dysfunction in porcine cells and that porMCL1 leads to increased mitochondrial respiration and therefore increased generation of ATP.

### MCL1 causes fusion of mitochondria, whereas FMDV causes mitochondrial fragmentation in porcine cells

**Fig 5:**
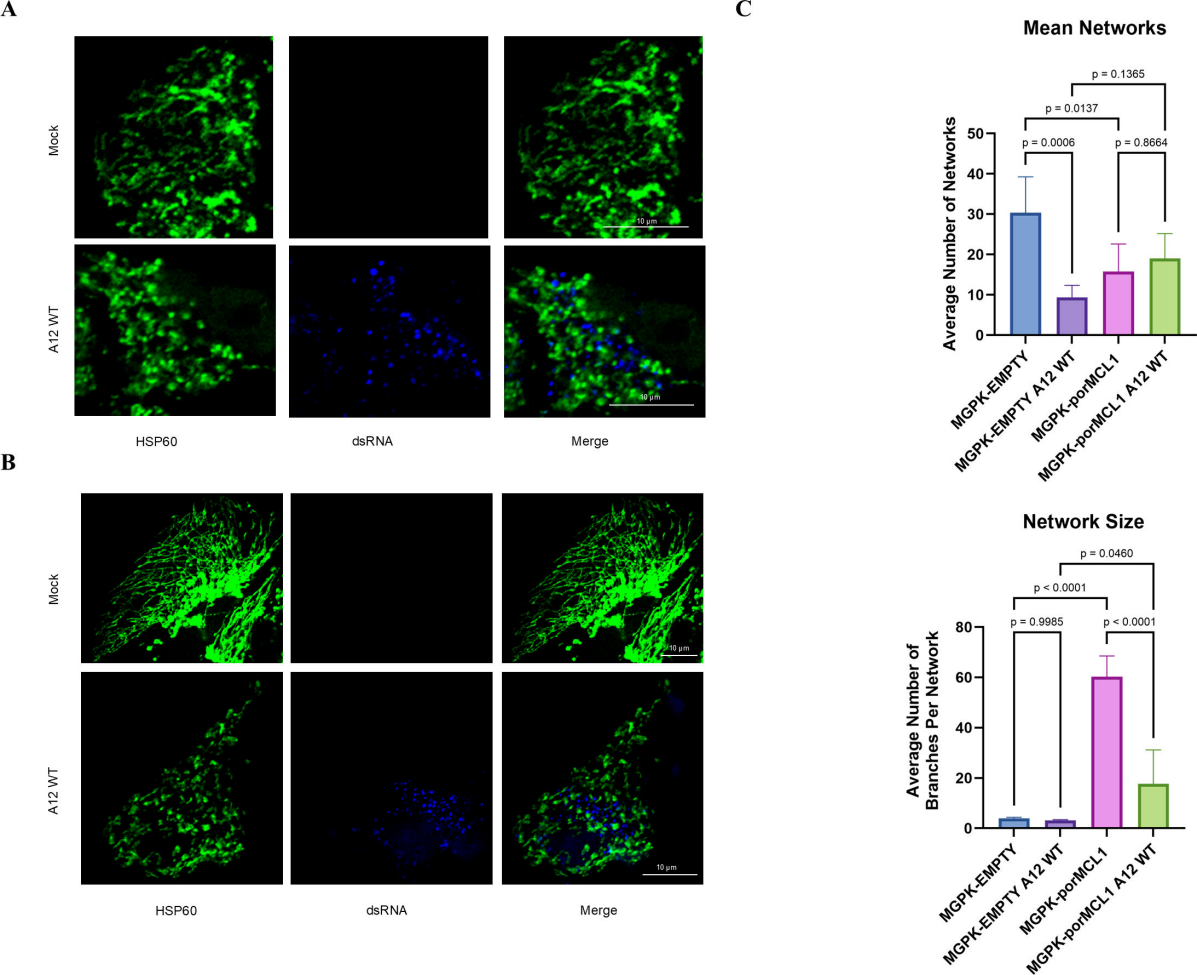
Mitochondrial morphology of porcine cells overexpressing MCL1. MGPK-EMPTY (**A**) and MGPK-porMCL1 (**B**) cells were either mock-infected or infected with FMDV A12 WT MOI: 5 and were fixed at 4 h post-infection. The proteins were visualized by indirect immunofluorescence. HSP60 was detected with a rabbit monoclonal antibody and then with Alexa Fluor 488-conjugated secondary antibody. RFP was an indicator for lentivirus-transduced cells (not shown). Double-stranded RNA (dsRNA) was detected with a mouse monoclonal antibody and then with Alexa Fluor 405-conjugated secondary antibody. The scale bar is representative of 10 µm. (**C**) Analysis of mitochondrial networks was done with Fiji (*n* = 5). The average number of networks graph is representative of all mitochondrial networks that contain one or more branches. The network size is indicative of an average number of branches per mitochondrial network. Statistical analysis was performed using one-way ANOVA with the Tukey *post hoc test*.

The results obtained above led us to investigate mitochondrial morphology as it has functional implications. When MGPK-αvβ6 cells transduced with the control SCRPSY-EMPTY lentivirus (RFP signal), mitochondrial cellular distribution (HSP60-green signal) exhibits various morphologies, including punctate and tubular mitochondria (Fig. 5A). However, upon FMDV infection, the mitochondria display fragmented morphology, and the tubular structures were rarely detected (Fig. 5A). In contrast, cells overexpressing porMCL1 displayed elongated morphology with increases in branching and networking (Fig. 5B). Although porMCL1-infected cells show some evidence of mitochondrial fragmentation, they do not exhibit the same level of mitochondrial punctate formation as the MGPK-EMPTY-infected cells (Fig. 5A), as some tubular mitochondria remain present. Further analysis of the microscopy images involved the quantification of two key features for each sample type: the average number of mitochondrial networks (defined as mitochondria with one or more branches) and the average number of branches per mitochondrial network (referred to as network size). Mitochondrial fission is characterized by decreased network size, as fragmented and punctate mitochondria have fewer branches. In contrast, increased mitochondrial fusion is characterized by a decrease in the number of networks and an increase in network size, as highly interconnected mitochondria have many branches. In uninfected MGPK-EMPTY samples, many mitochondrial networks were observed, but these networks were not highly branched, indicating a large population of small individual mitochondria. However, when those cells were infected, the number of mitochondrial networks decreased significantly, and the remaining networks that were present were poorly branched. This indicates that the mitochondria present were fragmented and lacked interconnectivity. In cells overexpressing MCL1, the number of networks was lower compared with the MGPK-EMPTY. However, the networks in these samples were highly branched, indicating the presence of longer and more interconnected mitochondrial networks. When these MCL1 overexpressing cells were infected, the number of mitochondrial networks did not significantly differ from the number of networks in infected MGPK-EMPTY cells. However, due to the increased network size, the mitochondrial networks existing in infected MGPK-porMCL1 cells had more branches, indicating more mitochondrial fusion comparatively (Fig. 5C). The confocal microscopy images corroborate the data generated from the mito stress test, establishing a connection between mitochondrial morphology and MCL1-dependent increase in mitochondrial respiration presumably linked to the observed anti-FMDV function.

### MCL1’s influence on mitochondrial dynamics is not due to mitochondrial calcium flux

**Fig 6:**
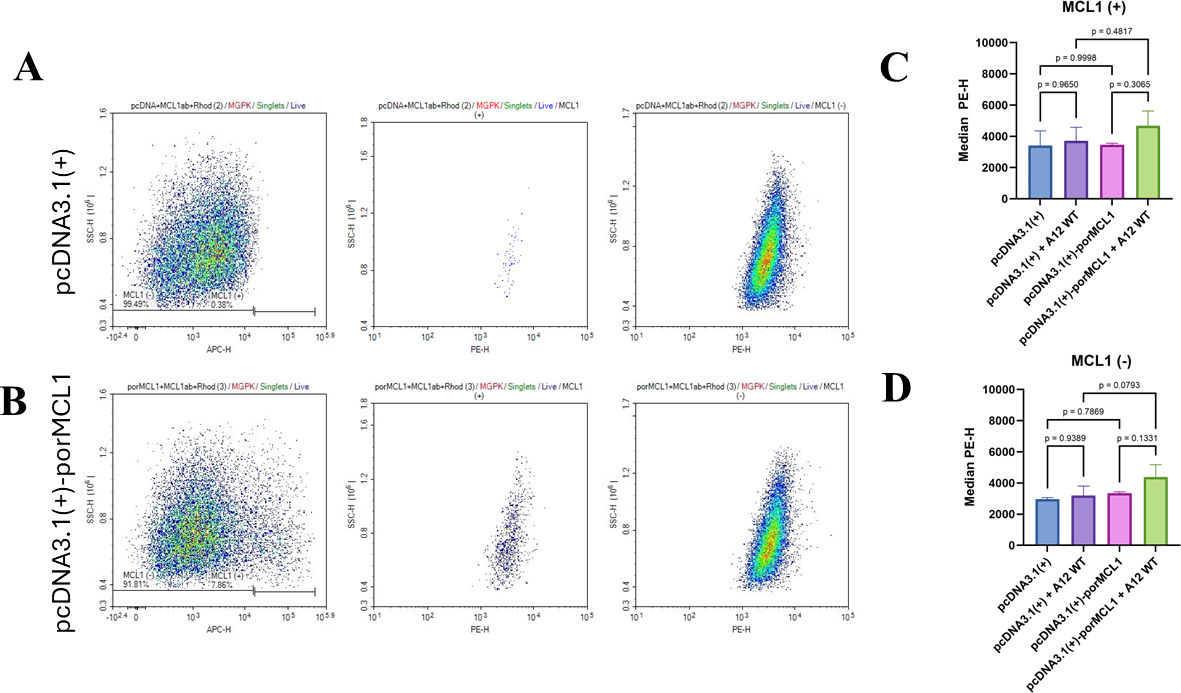
Mitochondrial calcium levels of porcine cells overexpressing MCL1. MGPK-αvβ6 cells were transfected with pcDNA3.1(+) or pcDNA3.1(+)-porMCL1 plasmids and then were mock-infected or infected with A12 WT MOI: 5 for 6 h. The cells were then stained with rhod 2-AM, viability dye, fixed, permeabilized, and finally stained with a monoclonal antibody for MCL1 that was conjugated to APC before running flow cytometry. (**A**) The figure represents the gating strategy for cells transfected with pcDNA 3.1(+), and (**B**) is for pcDNA 3.1(+)-porMCL1. The gating strategy employed segregation of cells overexpressing MCL1 (MCL1(+)) and cells endogenously expressing MCL1 (MCL1(-)). Rhod 2-AM was measured on the PE channel. (**C**) Representations of the median PE-H for both MCL1(+) and (**D**) MCL1(-) populations in the infected and uninfected samples. Statistical analysis was done using one-way ANOVA with the Tukey *post hoc* test.

As mitochondrial calcium has been shown to influence mitochondrial morphodynamics and that MCL1 affects mitochondrial calcium levels, we also sought to investigate this pathway. To assess mitochondrial calcium, we used Rhod 2-AM as an indicator, but due to the fluorescent spectral overlap with the RFP reporter gene present in the SCRPSY lentivirus, we decided to utilize plasmid transfection to overexpress MCL1. After gating for viable cells, we further categorize them into those overexpressing MCL1 and those expressing endogenous levels of MCL1 (Fig. 6A and B). In the MCL1 overexpression group of cells, there were no statistically significant differences in the levels of mitochondrial calcium when comparing infected versus non-infected cells (Fig. 6C). Similarly, cells expressing endogenous levels of MCL1 showed no significant differences when compared with the MCL1 overexpression group (Fig. 6D). The results demonstrate that the changes in mitochondrial dynamics associated with MCL1 are not due to mitochondrial calcium flux.

### MCL1 overexpression inhibits autophagy induced by FMDV infection

**Fig 7:**
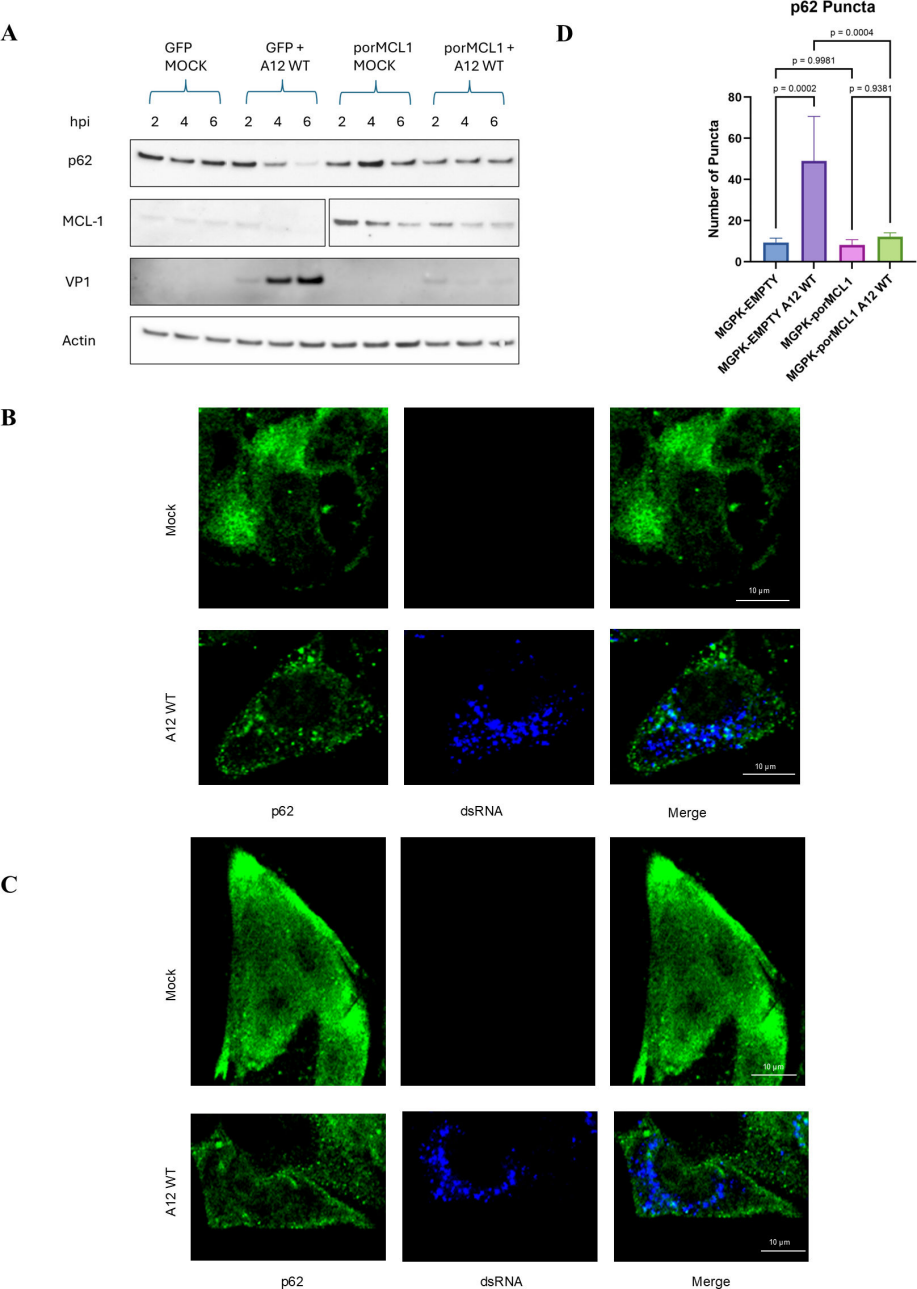
Analysis of autophagic flux with p62 puncta formation in porcine cells overexpressing MCL1. (**A**) Western blot analysis of lysates from MGPK-GFP and MGPK-porMCL1 cells subjected to a mock infection or infected with FMDV A12 WT at an MOI of 5 over a time course. The western blot was probed with antibodies against autophagic marker p62, MCL1, FMDV VP1, and actin as a loading control. (**B**) Indirect immunofluorescence imaging of p62 and dsRNA signals in MGPK-EMPTY cells compared with cells that were infected with FMDV A12 WT MOI: 5 for 6 h. (**C**) Indirect immunofluorescence imaging of MGPK-porMCL1 cells that were either mock-infected or infected for 6 h with FMDV A12 WT MOI: 5. The p62 protein was visualized using a polyclonal rabbit antibody and a secondary Alexa Fluor 488 antibody. The presence of dsRNA was visualized using a monoclonal antibody and a secondary Alexa Fluor 405 antibody. RFP was an indicator for lentivirus-transduced cells (not shown). The scale bar is representative for 10 µm. (**D**) Analysis of the number of p62 puncta was done with Fiji, and the graph represents the average number of puncta for each sample type (*n* = 5). Statistical analysis was performed using one-way ANOVA with the Tukey *post hoc* test.

**Fig 8:**
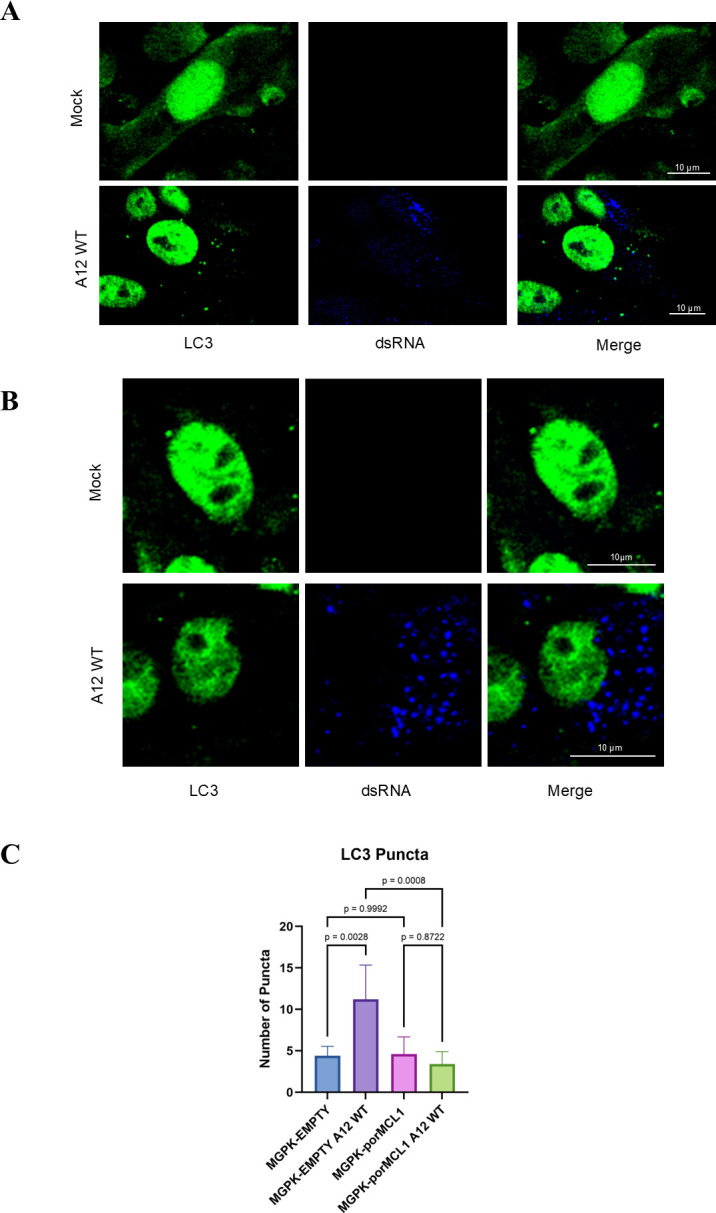
Analysis of autophagic flux with LC3 puncta formation in porcine cells overexpressing MCL1. MGPK-EMPTY (**A**) and MPGK-porMCL1 (**B**) cells were either mock-infected or infected with FMDV A12 WT MOI: 5 and were fixed 6 hours post-infection. The proteins were visualized using indirect immunofluorescence. LC3 was detected using a rabbit monoclonal antibody and a secondary antibody conjugated with Alexa Fluor 488. The presence of dsRNA was detected using a mouse monoclonal antibody and a secondary antibody conjugated with Alexa Fluor 405. RFP was an indicator for lentivirus-transduced cells (not shown). The scale bar is representative of 10 µm. (C) Analysis of the number of LC3 puncta was done with Fiji, and the graph represents the average number of puncta for each sample type (*n* = 5). Statistical analysis was performed using one-way ANOVA with the Tukey *post hoc* test.

Previous studies have shown that FMDV induces autophagy upon entry into the cell, as evidenced by the formation of LC3 puncta. Berryman et al. also demonstrated that one hallmark of autophagy induction was degradation of sequestosome-1 (p62/SQSTM1) over time when cells are infected with FMDV (26). Accordingly, when we measured p62 levels in a time course infection with cells overexpressing GFP, there was a decrease in p62 at 4 and 6 h post-infection. However, when the cells overexpressed porMCL1, the level of p62 remained relatively unchanged through the time course (Fig. 7A). Berryman et al. also observed the formation of p62 puncta in FMDV-infected cells by confocal microscopy (26). To further validate these findings, we used confocal microscopy to evaluate p62 distribution. In mock-infected, MGPK-EMPTY cells, p62 displayed a ubiquitous cytoplasmic expression pattern (Fig. 7B, *upper panels*). However, when infected with FMDV, discrete p62 puncta were clearly detected (Fig. 7B, *lower panels*). The same phenomenon was observed in cells that were starved of nutrients (Fig. S3). Similarly, mock-infected MGPK-porMCL1 cells showed a comparable cytoplasmic expression pattern of p62 with MGPK-EMPTY cells (Fig. 7C, *upper panels*). The difference lies in the FMDV-infected MGPK-porMCL1 expressing cells, as the numerous p62 puncta did not form and their expression remained ubiquitously in the cytoplasm (Fig. 7C, *lower panels*). The number of p62 puncta was statistically significantly higher in MGPK-EMPTY-infected cells, compared with any of the other sample groups (Fig. 7D). We next investigated the formation of LC3 puncta as another way to analyze autophagy. We were able to capture LC3 puncta by confocal microscopy, as evidenced in Fig. S5 with starved MGPK-αvβ6 cells. As previously reported for other cell lines, MGPK-αvβ6 cells also display nuclear localization of LC3 (33). Analysis of LC3 puncta formation demonstrated a similar pattern as the p62 microscopy images, where there is a statistically significantly higher number of LC3 puncta in infected MGPK-EMPTY cells compared with the other samples (Fig. 8A and C). In a similar fashion, the LC3 puncta formation was not statistically different in MGPK-porMCL1 cells, even when infected with FMDV (Fig. 8B and C). Together, western blot and the lack of increased p62 and LC3 puncta formation in the infected MGPK-porMCL1 demonstrate an inhibition of autophagy by MCL1. As autophagy is necessary for FMDV infection, these results demonstrate that MCL1-dependent inhibition of autophagy may have also contributed to its antiviral function.

### MCL1 overexpression does not increase the expression of ISG15 transcripts

**Fig 9:**
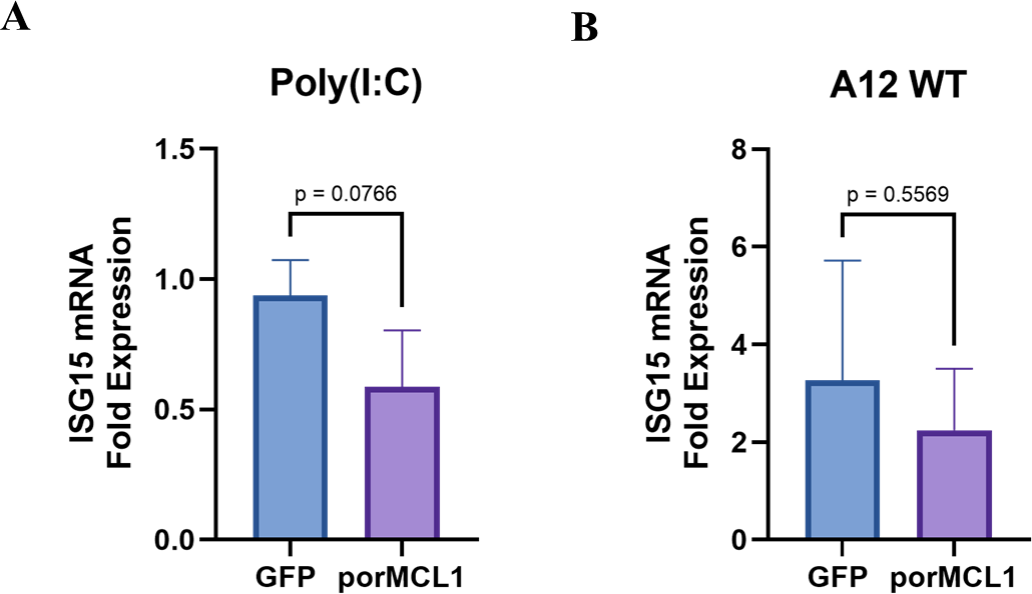
Expression of ISG15 mRNA in response to stimulation with poly(I:C) or A12 WT infection. Total RNA was isolated in MGPK-GFP and MGPK-porMCL1 cells that were either treated with poly(I:C) (**A**) or infected with A12 WT MOI: 5 (**B**) to analyze the mRNA fold expression of ISG15 at 6 h post-infection by real-time RT-PCR. Porcine HRP-T was used as an internal control, and all the results were expressed as a relative fold increase in gene expression with respect to mock-treated cells (*n* = 3). Statistical analysis was performed using Student’s *t*-test.

To determine if MCL1 overexpression causes stimulation of the innate immune response due to its role in mitochondrial dynamics and autophagy, the expression of ISG15 was analyzed by real-time RT-PCR. ISG15, a strongly induced ISG in response to type I IFN, poly(I:C), and other stressors, is known to have inhibitory activity against FMDV. MGPK-GFP or porMCL1 cells were treated with either poly(I:C), mock-infected, or infected with A12 WT. Poly(I:C) was used as a control for stimulating the RLR response to induce ISG transcription, as wild-type FMDV can interfere with transcription of ISG antiviral responses (34). Analysis of ISG15 mRNA fold induction following both treatments demonstrated that there was no statistically significant difference between MGPK-GFP and MGPK-porMCL1 cells (Fig. 9). This finding indicates that the overexpression of MCL1 does not enhance the activation of the innate immune response. Therefore, the antiviral activity of MCL1, which is influenced by its roles in mitochondrial dynamics and autophagy, cannot be ascribed to the activation of the innate immune response.

## DISCUSSION

One of the main tactics of FMDV to evade host immune responses is through the suppression of IFN. The onset of IFN generation leads to the induction of various ISGs that have antiviral activity. From previous research, ISGs like ISG15, PKR, etc, have been proven to be inhibitory against FMDV replication (34, 35). In this work, we described a high-throughput screen that has identified novel ISGs that inhibit FMDV replication (5). This screening approach offered two key advantages: multiple genes could be effectively tested at once, and an FMDV replicon could be used instead of a whole virus, thus preventing the need for a costly BSL-3 laboratory. From this screen, we identified MCL1 as an ISG that is inhibitory to FMDV replicon replication. Validation of this screen by overexpression of MCL1 in porcine cells proved that it does indeed inhibit FMDV viral replication as early as 6 h post-infection (Fig. 1A). With these data, we tried to elucidate the mechanism of the antiviral function of MCL1.

Due to the various studies indicating that FMDV infection or specific FMDV proteins induce apoptosis, we initially hypothesized the anti-apoptotic function of MCL1 might serve as its antiviral mechanism. However, there are caveats with these previous apoptosis studies with FMDV. Many of them do not utilize wild-type virus, whereas others do not use physiologically relevant cell lines or natural hosts for FMD (8–12). Multiple studies also only analyze the detection of apoptosis after 24 h post-infection, whereas we see the antiviral activity of MCL1 as early as 6 h post-infection. When we tried to mimic the inhibition of apoptosis by treating MGPK-αvβ6 cells with the caspase inhibitor Q-VD-OPH, we observed no significant differences in viral titer at any time point tested (Fig. 2B). These results indicate that blocking apoptosis is not the antiviral function of MCL1, as we would have expected to see some difference in viral titers at early time points between 2 and 6 h post-infection.

The next functional pathway explored was MCL1’s regulation of the cell cycle. One study examining FMDV’s influence on cell cycle demonstrated that at 24 h post-infection, BHK-21 cells had more viral RNA copies and more cells containing the 3D polymerase of FMDV in cells in the G2/M phase. The authors also demonstrated that blocking cells at the G2/M phase produced more viral progeny after determining viral titers at 20 h post-infection (16). In the context of MGPK-αvβ6 cells, we did not see this shift toward G2/M phase at 6 h post-infection (Fig. S2). Furthermore, overexpression of MCL1 did not cause an arrest in the cell cycle, implying that its role in the cell cycle is not involved with its antiviral function (Fig. 3B).

As the subcellular localization of MCL1 is in the mitochondria, we thought one of the more likely mechanisms of its antiviral function would be the manipulation of mitochondrial dynamics. Mitochondrial dynamics are important regulatory pathways for viruses to manipulate, as mitochondria are involved in various cellular processes such as metabolism, apoptosis, calcium homeostasis, oxidative stress, and innate immune signaling. Therefore, mitochondrial function and shape are very important for dictating antiviral responses. There are only a few reports on the involvement of mitochondria or mitochondrial-related proteins with FMDV. One study demonstrated that mitochondrial damage was induced over the course of infection with FMDV, as determined by increases of mitochondrial ROS (specifically superoxide), mitochondrial calcium, and mitochondrial pore opening. They also reported that DNA was released from the mitochondria by FMDV 2B protein (21). Two other reports demonstrated that FMDV VP1 and VP3 inhibited the function of MAVS (22, 36). These reports indicate that FMDV causes mitochondrial dysfunction. Studies done with MCL1 show various effects on mitochondrial function and morphology. One study showed that deletion of MCL1 leads to increased ROS levels, decreased ATP production, decreased OCR, and reduction of mitochondria with tubular morphology. The authors also demonstrated that MCL1 is needed for the assembly of ATP synthase oligomers, which is why OCR and ATP production decreased with MCL1 deletion (19). Another study has demonstrated that MCL1 stabilizes the mitochondrial fusion protein, OPA1, leading to mitochondrial fusion (37). These findings imply that MCL1 is needed for increased oxidative phosphorylation to generate ATP and that MCL1 can contribute to mitochondrial fusion.

From our analysis of mitochondrial respiration, we discovered that FMDV does indeed cause mitochondrial dysfunction due to the decreased basal respiration and ATP production. Conversely, overexpression of MCL1 increases basal respiration and ATP production in cells (Fig. 4B and C), although not under FMDV infection conditions. Additionally, MCL1 allowed for increased coupling of oxygen to ATP production under infection conditions (Fig. 4D). The data from these experiments reveal that FMDV causes mitochondrial dysfunction to significantly reduce oxidative phosphorylation. The increase in oxidative phosphorylation with overexpression of MCL1 corroborates previous data, which show that depletion of MCL1 decreases ATP production from oxidative phosphorylation (19). In addition, MCL1 expression leads to more oxygen being coupled to ATP production rather than being coupled to proton leak during the stress conditions of an FMDV infection. Increased proton leak is usually associated with oxidative stress, cellular dysfunction, and decreased production of ATP, and these processes could be utilized to benefit viral replication (38). Further studies need to be done to elucidate the benefit of proton leak and decreased oxidative phosphorylation with FMDV replication.

This experiment also sheds light on the theory that FMDV might be inducing the “Warburg effect.” The “Warburg effect” was first defined in the context of tumor cells, which switch from oxidative phosphorylation to glycolysis to generate ATP in a rapid manner (39). Due to the nature of FMDV as a rapidly replicating virus, quick generation of ATP may be needed for its replication. However, further research needs to be done to establish whether glycolysis or other pathways, like fatty acid oxidation or glutaminolysis, are used to generate ATP. Subverting metabolism may not only be important for viral replication but also for subverting immune responses. T cell mitochondrial metabolism is instrumental in determining responses of T cells, such as effector or memory responses, as energetic demand needs to be met depending on the type of response (40). Therefore, further study into mitochondrial metabolism can help us design better vaccines by fine-tuning metabolism-associated T cell responses.

From the analysis of mitochondrial respiration, we could theorize what the impact of FMDV and MCL1 was on mitochondrial morphology. Usually, a decrease in oxidative phosphorylation is linked to mitochondrial fragmentation, and an increase in oxidative phosphorylation is linked to mitochondrial fusion (41). From analyses using confocal microscopy, we were able to see that overexpression of MCL1 did indeed induce highly networked and branched mitochondria. In cells that were infected with FMDV, we found that there was mitochondrial fragmentation and the formation of punctate mitochondria. The cells overexpressing MCL1 and infected with FMDV also demonstrated fragmented mitochondria; however, there were fewer punctate mitochondria and more branched mitochondrial networks comparatively (Fig. 5). Previous works have established that MCL1, which is located in the inner mitochondrial matrix, leads to mitochondrial fusion, and this may explain the phenotype we are seeing in cells overexpressing MCL1^19^.

Due to the impact of FMDV and MCL1 on mitochondrial dynamics, we wanted to discern if these manipulations are due to changes in mitochondrial calcium flux. As mentioned before, FMDV has been shown to increase mitochondrial calcium over time (21). MCL1 has also been shown to increase mitochondrial calcium, leading to an increase in mitochondrial ROS, but only when interacting with voltage-dependent anion channel (VDAC) on the outer membrane matrix (42). When we analyze mitochondrial calcium levels in cells overexpressing MCL1, we do not see statistically significant differences in mitochondrial calcium levels when compared to cells that do not overexpress MCL1. Even after infection, there was no significant effect on mitochondrial calcium levels (Fig. 6). The lack of differences in mitochondrial calcium levels indicates that the effect on mitochondrial dynamics was not due to calcium flux.

In this study, we also investigated the influence MCL1 has on the induction of autophagy, as previous work has shown that FMDV induces autophagy. O’Donnell et al. demonstrated co-localization of FMDV viral proteins with autophagosomes, in which it was demonstrated that there are higher viral yields with the induction of autophagy (27). Berryman et al. described that FMDV induces autophagosome formation at early time points during cell entry (26). MCL1 has also been shown to inhibit autophagy by inhibiting the activity of Beclin-1, a protein involved in inducing autophagy. MCL1 and Beclin-1 are both regulated by the deubiquitinase USP9X. When MCL1 binds to USP9X to become deubiquitinated, Beclin-1 gets ubiquitinated instead and targeted for proteasomal degradation, blocking autophagy. With this knowledge in mind, we examined autophagy through the marker p62 in MCL1 overexpressing cells to determine if MCL1 has a regulatory role. A study previously conducted on MCL1’s regulatory role on autophagy revealed that overexpression of MCL1 leads to an increase in expression of p62 and reduced p62 degradation in starvation conditions (43). We were able to replicate these findings, showing that FMDV infection induces autophagy, as evidenced by the degradation of p62 autophagic marker over time and formation of p62 puncta in MGPK-EMPTY cells (Fig. 7A, B, and D). However, it is important to note that puncta formation was only evaluated at 6 hpi and not throughout a time course. Overexpression of MCL1 did not lead to p62 degradation or p62 puncta formation, meaning that autophagy was inhibited (Fig. 7A, C, and D). LC3 puncta formation was also demonstrated in MGPK-EMPTY-infected cells and was notably lower in MCL1 overexpressing cells (Fig. 8). These results corroborated the previous study’s conclusions regarding MCL1’s role in the inhibition of autophagy. The inhibition of autophagy could be one of the main drivers of the MCL1-dependent inhibition of FMDV replication by MCL1

Several studies on FMDV have explored the role of proteins involved in autophagy to dampen innate immune responses. These studies showed that FMDV utilizes autophagy machinery to degrade other proteins like G3BP1, YTHDF2, and HDAC8, ultimately degrading various elements along the innate immune signaling axis (28–30). In addition, research has shown that mitochondrial fusion could increase RLR signaling through MAVS (44). However, our analysis has determined that overexpression of MCL1 does not lead to an increased innate immune response (Fig. 9). Therefore, increased mitochondrial fusion and inhibition of autophagy do not induce an IFN response, and MCL1’s antiviral mechanism directly impacts FMDV replication. These findings highlight an important aspect of the host antiviral response, as ISGs like MCL1 can have a direct effect on viral replication without stimulating a persistent IFN response that could result in damaging inflammation or immune dysfunction.

**Fig 10:**
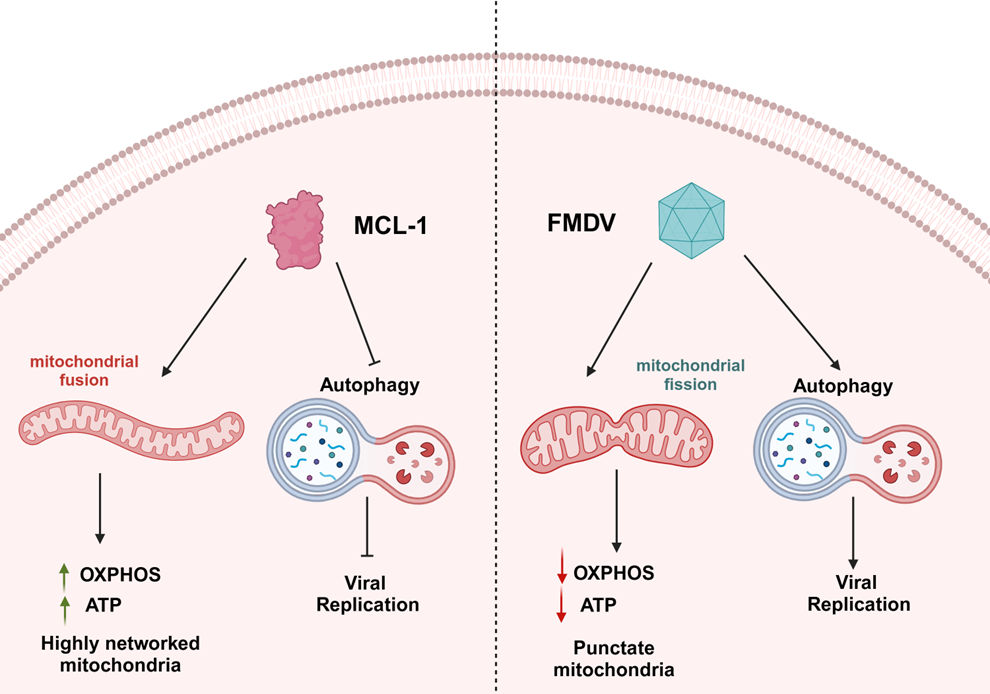
Effect of MCL1 and FMDV on mitochondrial dynamics and autophagy. This model illustrates the contrasting effects of MCL1 overexpression and FMDV infection on mitochondrial dynamics and autophagy in porcine cells. MCL1 overexpression in porcine cells leads to increased mitochondrial fusion, leading to increased oxidative phosphorylation, resulting in increased ATP production and highly networked mitochondria. On the other hand, FMDV leads to mitochondrial fragmentation, leading to decreased oxidative phosphorylation and associated ATP production, as well as the formation of punctate mitochondria. Additionally, FMDV triggers autophagy and exploits the autophagic machinery to facilitate its viral replication. MCL1, by inhibiting autophagy, reduces the availability of this machinery for viral replication, ultimately leading to decreased FMDV replication. Created in BioRender.com

The findings of this work provide valuable insights into the molecular mechanisms that FMDV manipulates to facilitate its replication. We have established that MCL1 has an antiviral function against FMDV replication, primarily through its regulation of mitochondrial dynamics and autophagy (Fig. 10). There are limitations in this study, including the focus on the porcine host and confining ourselves to *in vitro* experiments only. Also, all experiments were done at early time points, whereas animals with FMD can sustain infection for multiple days. However, this study serves as a jumping-off point for exploring important aspects of the virus host interactions of FMDV. Future studies should focus on identifying which specific FMDV viral proteins manipulate mitochondrial morpho-dynamics. With current FMD vaccine strategies, an adaptive protective immune response requires 5–7 days, leaving a window of time where vaccinated animals are susceptible to infection with FMDV infection (45). Therefore, biotherapeutics and adjuvants can be potentially engineered to specifically target these autophagic and mitochondrial pathways that FMDV influences. A combination of these new therapeutic and current vaccine strategies can help protect animals against FMDV infection before the onset of protective immunity. In addition, autophagy and mitochondrial function are very important in dictating the function, polarization, and responses of immune cell subsets (46, 47). Knowledge of the effects of these specific pathways on the immune response could help design better vaccine strategies to increase protective humoral and cellular immune responses. In terms of vaccine design, a deeper understanding of FMDV viral proteins involved in subverting mitochondrial dynamics and initiating autophagy could aid in designing live-attenuated vaccines. Mutations or deletions in the FMDV viral genome involved with these pathways could lead to attenuated profiles that could elicit an immune response without causing significant clinical illness in animals. Altogether, incorporating these pathways into rational vaccine design could enhance strategies that leverage innate immunity and activate adaptive immunity.

## MATERIALS AND METHODS

### Cells and viruses

HEK293T cells were used to grow lentiviral constructs and were maintained with Dulbecco’s MEM (DMEM) containing 10% fetal bovine serum (FBS), glutamine, non-essential amino acids (NEAA), and antibiotics (AA). MGPK-αvβ6 cells were used to generate stable cell lines to be used in further assays and were maintained in DMEM containing 10% FBS, non-essential amino acids, and antibiotics (48). BHK-21 cells were used to titrate FMDV by plaque assay. They are maintained with MEM containing 10% FBS, 10% tryptose phosphate broth, non-essential amino acids, and antibiotics. The FMDV virus strain used for infection was A12 wild-type (A12 WT). The lentivirus constructs used for generating stable cell lines were SCRPSY-ISG-TagRFP.

### Generation of lentivirus

HEK293T cells were seeded in a T-25 at around 80% confluency on the day of transfection. To generate the SCRPSY-ISG-TagRFP lentiviruses, 293T cells were transfected with plasmid DNA at the ratio of 5 µg SCRPSY-ISG-TagRFP:1 µg pTRIP.CMV.IVSb.NLGP.ires.TagRFP: 0.2 µg pTRIP.CMV.IVSb.VSV-G.ires.TagRFP, using Xtremegene9 (Sigma). Supernatants were collected daily, and the 48, 72, and 96 h supernatants were pooled together and aliquoted. The SCRPSY constructs used for the experiments were SCRPSY-huMCL1, SCRPSY-porMCL1, SCRPSY-GFP, and SCRPSY-EMPTY.

### Generation of stable cell lines

MGPK-αvβ6 cells were seeded in a T-25 to be around 80% confluent on the day of transduction. Media was changed to transduction media where the final concentrations of polybrene and HEPES were 10 μg/mL and 20 mM, respectively, in complete MGPK-αvβ6 media; 2 mL of lentivirus supernatant was added dropwise to the cells, and the T-25s were incubated at 37°C overnight. The next day, the media was changed to complete MGPK-αvβ6 medium. Two days post-transduction, the cells would reach maximum RFP expression, and then, the media was changed to complete MGPK-αvβ6 medium, including 5 μg/mL puromycin. After 2 days of puromycin selection, the cells were split and passaged into another T-25 and maintained with 1 µg/mL of puromycin.

### Infection of stable cell lines

MGPK-αvβ6 cells transduced with lentivirus were seeded at 3 × 10^5^ cells/well in a 24-well plate to be incubated overnight at 37°C. The next day, they were infected with FMDV A12 WT at a multiplicity of infection (MOI) of 5. After 1 h of incubation with the virus, the cells were washed with MES buffer (pH 5.5), DMEM, and then left with 1 mL of DMEM with NEAA and AA. At 6 h post-infection, plates were frozen at –70 ° C. At the same time point, the replicates were lysed and collected for western blot so that protein expression of FMDV VP1/VP3, actin, and MCL1 could be evaluated. Frozen supernatants were titered by plaque assay on BHK-21 cells.

### Western blot

Cell lysates were collected with G Lysis buffer (100 mM Tris pH 8.0, 1 mM EDTA, 50 mM NaCl, 1% NP40). The samples were separated through an SDS-PAGE gel and transferred onto a nitrocellulose membrane. The membrane was probed with various primary antibodies, including MCL1 (CST, 4572S), actin (Sigma, A1978), VP1 (developed at PIADC), and p62 (Proteintech, 18420–1-AP), and PARP (CST, 9541). The membrane was then probed with either of these horseradish peroxidase-conjugated secondary antibodies (Invitrogen): goat anti-rabbit Immunoglobulin G (IgG) or goat anti-mouse IgG. The blots were then incubated with the reagents from an ECL chemiluminescence kit (Bio-Rad) and imaged with the chemiluminescent imager Azure R Imager c300.

### Treatment with Pan-caspase inhibitor Q-VD-OPH

MGPK-αvβ6 cells in 24-well plates were treated with either DMSO, 20 µM of Q-VD-OPH, 1 µM of Staurosporine, a combination of 1 µM of Staurosporine and 20 µM of Q-VD-OPH, A12 WT MOI: 5, or A12 WT MOI: 5 combined with 20 µM of Q-VD-OPH. Western blot lysates were collected at 2, 4, and 6 h post-infection. Supernatants were frozen at the above time points from three extra wells of the two groups infected with FMDV. These supernatants were later titered by plaque assay on BHK-21 cells. The western blot lysates were probed for VP1, actin, and PARP.

### Cell cycle analysis by flow cytometry

Transduced MGPK-αvβ6 cells were seeded in six-well plates at a density of 1 × 10^6^ cells per well and incubated overnight at 37°C. Cells were then trypsinized and resuspended in MGPK-αvβ6 complete medium. After pelleting once and washing once with phosphate-buffered saline (PBS), cells were pelleted again and resuspended in 300 µL of PBS. Cells were then vortexed as 700 µL of chilled 100% ethanol was added in a dropwise manner. Fixed cells were then incubated at 4°C for 1 h and then stored at –20°C. On the day of flow cytometric analysis, the cells were pelleted and washed twice with PBS. After counting, 1 × 10^6^ cells were aliquoted and resuspended in staining solution consisting of 0.1% Triton-X in PBS and 10 μg/mL of DAPI. After incubation at room temperature in the dark, cells were analyzed on the Agilent Novocyte 3000. Data were analyzed with Agilent Novoexpress software. The percentage of cells in each cell cycle phase was gated based upon the expression of GFP or RFP signal to capture only transduced cells.

### Seahorse Mito Stress assay

Prior to performing the Agilent Seahorse XFp Mito Stress assay, the optimal cell density was determined to be 20,000 cells per well using the Agilent Seahorse XFp ATP rate assay. The day prior to assay, either the MGPK-GFP or MGPK-porMCL1 cells were seeded in the Seahorse XFp Cell Culture Miniplate according to the manufacturer’s instructions using cell culture growth medium. The sensor cartridge was also hydrated and placed at 37°C in a non-CO_2_ incubator overnight. On the day of the assay, three of the wells from each plate were infected with FMDV A12 WT MOI: 5 for 3 h. During that time, Seahorse XF DMEM was warmed to 37°C and prepared with 15 mM glucose, 4 mM glutamine, and 1 mM pyruvate. One hour prior to loading the plate into the Agilent Seahorse HS Mini, the cell culture miniplate was washed with Seahorse XF DMEM according to the manufacturer’s instructions and incubated at 37°C in a non-CO_2_ incubator to allow for degassing. The sensor cartridge was also hydrated with XF calibrant for 45 min at 37°C in a non-CO_2_ incubator prior to loading into the machine. The compounds oligomycin, FCCP, and rotenone/antimycin A were resuspended and loaded into the sensor cartridge ports to yield final treatment concentrations of 1.5, 1.5, and 0.5 µM, respectively. After calibration of the sensor cartridge, the cell culture miniplate was run on the Agilent Seahorse HS Mini with the sensor cartridge injecting the compounds at the indicated time points. Oxygen consumption rate (OCR) and extracellular acidification rate (ECAR) were measured three times before the first compound was injected to establish a baseline, and rates were also measured three times after the addition of each compound. The OCR measurements were then normalized based on cell number for statistical analysis using the Agilent Seahorse Analytics.

### Immunofluorescence assay

MGPK -αvβ6, either transduced with SCRPSY-porMCL1 or SCRPSY-EMPTY, was seeded on 24-well plates on coverslips at a cell density of 8 × 10^4^ cells per well. Cells were incubated overnight and either infected with FMDV A12 WT MOI: 5 or mock-infected for 4–6 h before fixation. The coverslips were fixed with 4% paraformaldehyde resuspended in PBS for 20 min and then washed three times with PBS. The cells were then permeabilized with 0.5% Triton-X for 5 min and washed three times with PBS. The coverslips were blocked for 1 hour with 5% bovine serum albumin (BSA) resuspended in PBS and then incubated overnight with primary antibody resuspended in the blocking solution at 4°C. The next day, cells were washed three times with PBS, incubated with secondary antibody at room temperature in the dark for 1 h. After washing with PBS three times, the coverslips were mounted onto microscope slides with Prolong Glass Antifade Mountant. The primary antibodies used were HSP60 (CST, D6F1), dsRNA (EMD Millipore, MABE1134), p62 (Invitrogen, 18420–1-AP), and LC3A/B (CST, D3U4C). The secondary antibodies were Goat anti-rabbit IgG Alexa Fluor 488 and Goat anti-mouse Alexa Fluor 405 (Invitrogen). All slides were captured using a confocal microscope Zeiss LSM710 at either 63× or 100× magnification. Images were visualized using ZEN 3.0 software, Blue Edition. Transduced cells displaying RFP signal were used for analysis (not shown). The software Fiji was used to process the images to determine the number of puncta, and the plugin Analyze Skeleton was used to analyze mitochondrial networks (49, 50).

### Mitochondrial calcium analysis by flow cytometry

MGPK-αvβ6 cells were seeded at 1 × 10^6^ cells per well and incubated overnight at 37°C. The next day, the cells were transfected using Lipofectamine 2000 (Invitrogen) with either 2 µg of pcDNA3.1(+) or pcDNA3.1(+)-porMCL1 plasmid. After overnight incubation at 37°C, cells were either infected with A12 WT MOI: 5 or mock-infected for 4 h. The cells were then trypsinized, and 5 × 10^5^ cells were resuspended in calcium staining solution. This solution consisted of DMEM, NEAA, AA, 0.02% Plurionic F-127 (Invitrogen), and 2.5 µM of Rhod 2-AM (Abcam). After incubation in the dark at 37°C for 30 min, the cells were washed twice with calcium-free PBS. The cells were incubated at 37°C for another 30 min to allow for the de-esterification of Rhod 2-AM. After the de-esterification process, cells were stained with viability dye (Invitrogen), fixed and permeabilized (BD Biosciences), and then stained with antibody against MCL1 that was conjugated with APC (CST, D2W9E). After more wash steps, cells were run on the Agilent Novocyte 3000. Data were analyzed with Agilent Novoexpress software.

### Real-time RT-PCR

Total RNA was isolated from MGPK-GFP and MGPK-porMCL1 cells that were mock-infected, infected with A12 WT MOI: 5, or treated with 2.5 µg/mL poly I:C at 4 h post-infection using the RNAeasy Mini kit (Qiagen); 1 µg of total RNA treated with DNase I (Neb) was synthesized into cDNA using Moloney murine leukemia virus reverse transcriptase (Invitrogen), and random primers. Relative expression of the ISG ISG15 from the total cDNA was determined by qPCR with PerfeCTa SYBR Green FastMix Low ROX with specific primers described for porcine ISG15 (51). The expression of porcine HRPT1 was used to normalize the expression level of ISG15. cDNA was amplified for 40 cycles using ABI Prism 7500, and relative expression was quantified using the 2^−ΔΔCT^ method.

## SUPPLEMENTAL MATERIAL

Figure S1 jvi.00581-25-s0001.TIF siRNA knockdown of MCL1.

Figure S2 jvi.00581-25-s0002.TIF Cell cycle analysis of MGPK-αvβ6 cells.

Figure S3 jvi.00581-25-s0003.TIF Mito stress test of MGPK-αvβ6.

Figure S4 jvi.00581-25-s0004.TIF p62 immunofluorescence of MGPK-αvβ6.

Figure S5 jvi.00581-25-s0005.tif LC3 immunofluorescence of MGPK-αvβ6.

Supplemental material jvi.00581-25-s0006.docx Supplemental methods and figure legends.

Table S1 jvi.00581-25-s0007.TIF ISG replicon screen results.

ASM does not own the copyrights to Supplemental Material that may be linked to, or accessed through, an article. The authors have granted ASM a non-exclusive, world-wide license to publish the Supplemental Material files. Please contact the corresponding author directly for reuse.
