# Supplementary figures and images for: Interferon-stimulated gene MCL1 inhibits foot-and-mouth disease virus replication by modulating mitochondrial dynamics and autophagy
Source: J Virol. 2025 Jun 4;99(7):e00581-25. doi: 10.1128/jvi.00581-25 (PMC12282159; doi:10.1128/jvi.00581-25)

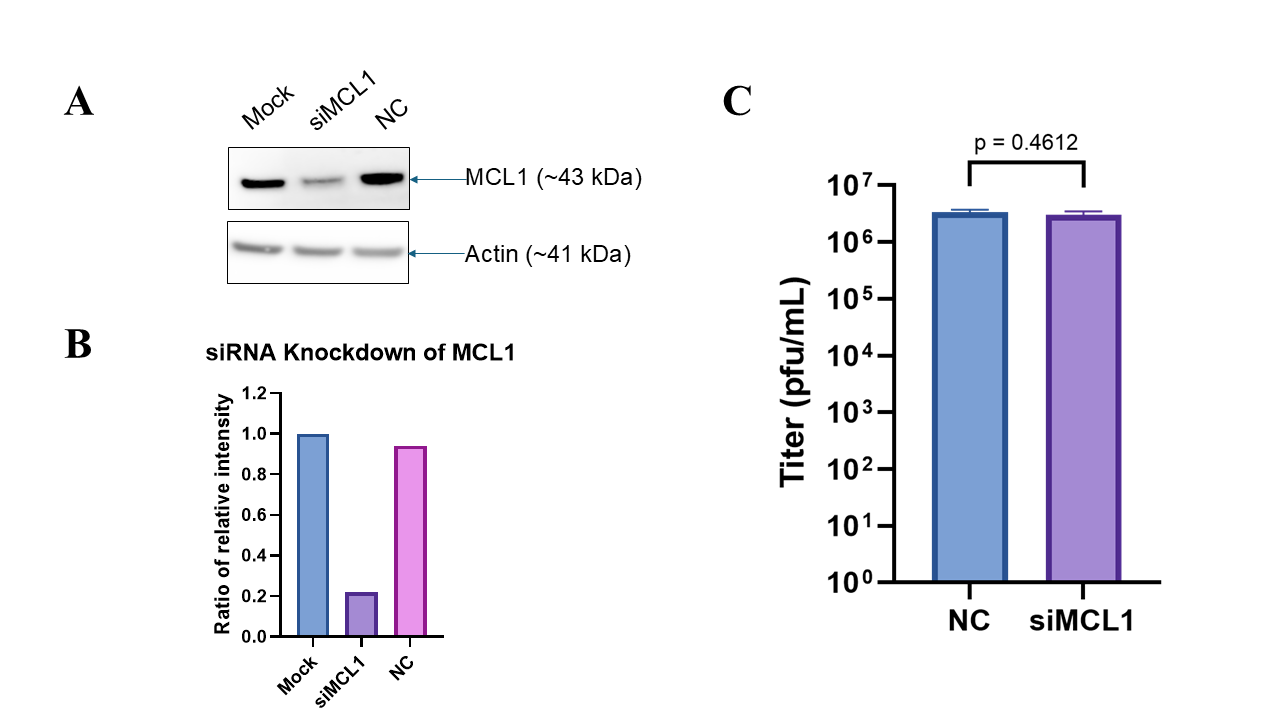

Supplement: Figure S1 — siRNA knockdown of MCL1. [file jvi.00581-25-s0001.tif]

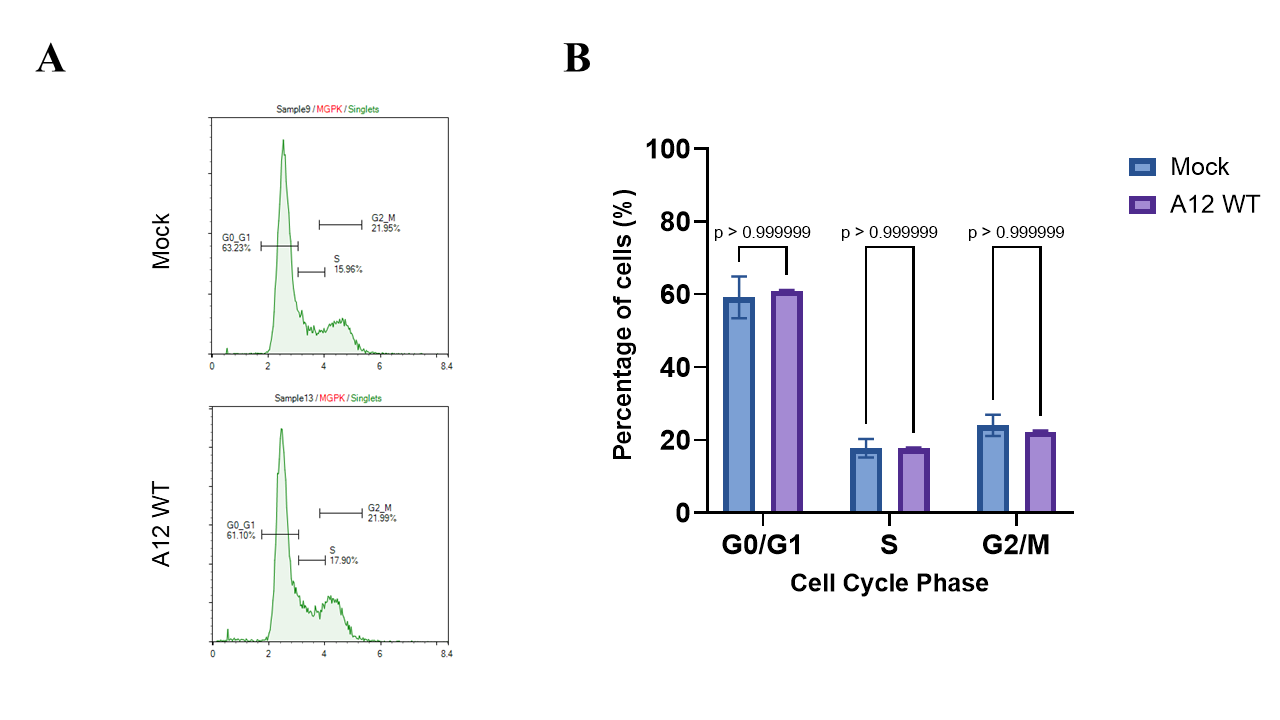

Supplement: Figure S2 — Cell cycle analysis of MGPK-αvβ6 cells. [file jvi.00581-25-s0002.tif]

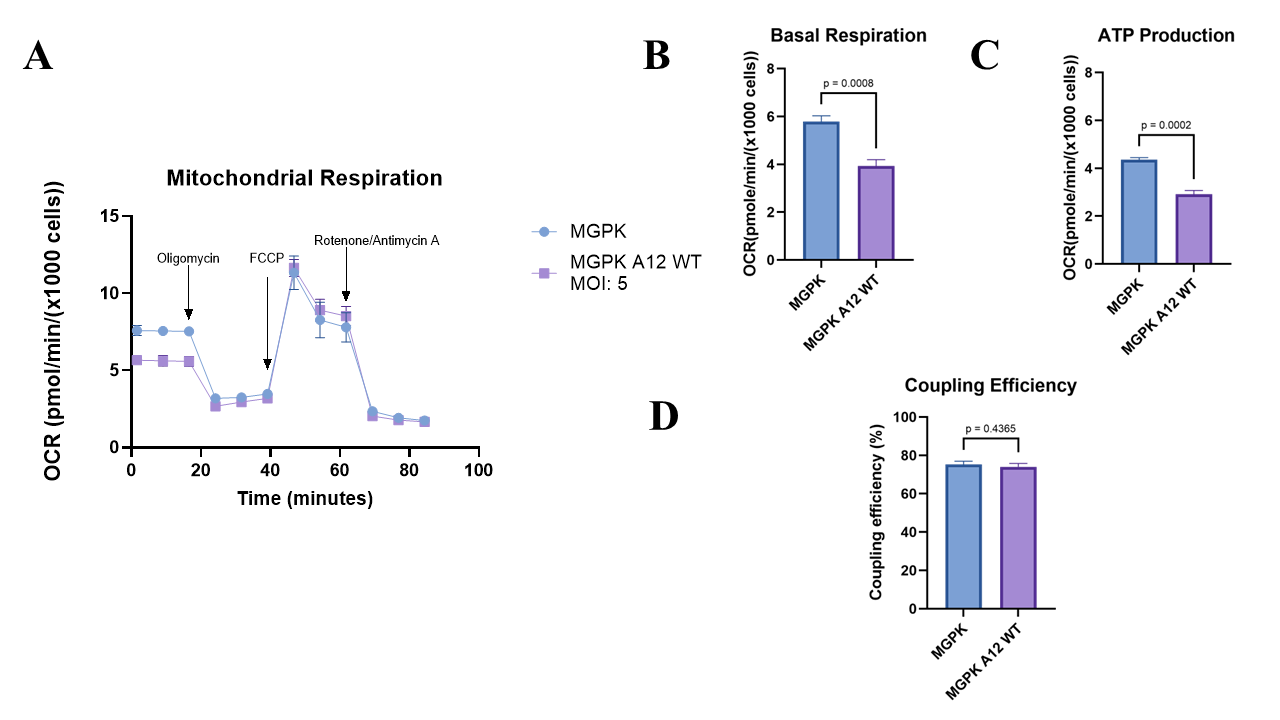

Supplement: Figure S3 — Mito stress test of MGPK-αvβ6. [file jvi.00581-25-s0003.tif]

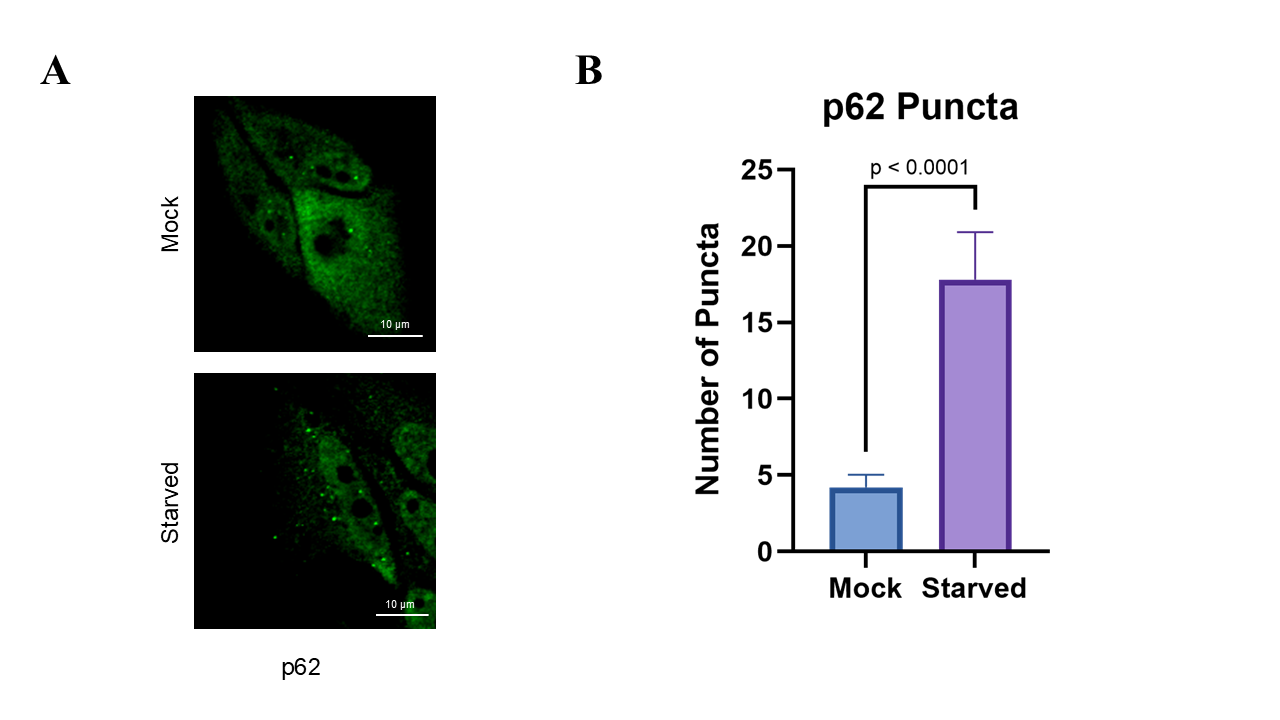

Supplement: Figure S4 — p62 immunofluorescence of MGPK-αvβ6. [file jvi.00581-25-s0004.tif]

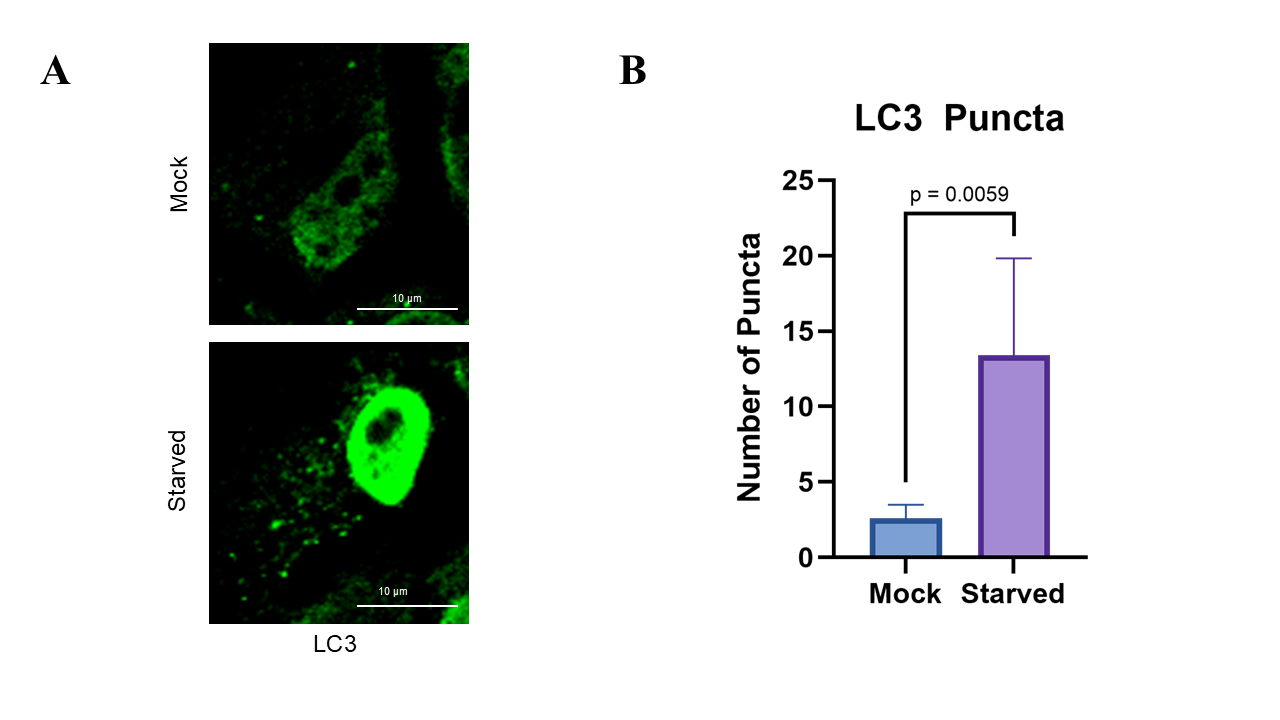

Supplement: Figure S5 — LC3 immunofluorescence of MGPK-αvβ6. [file jvi.00581-25-s0005.tif]

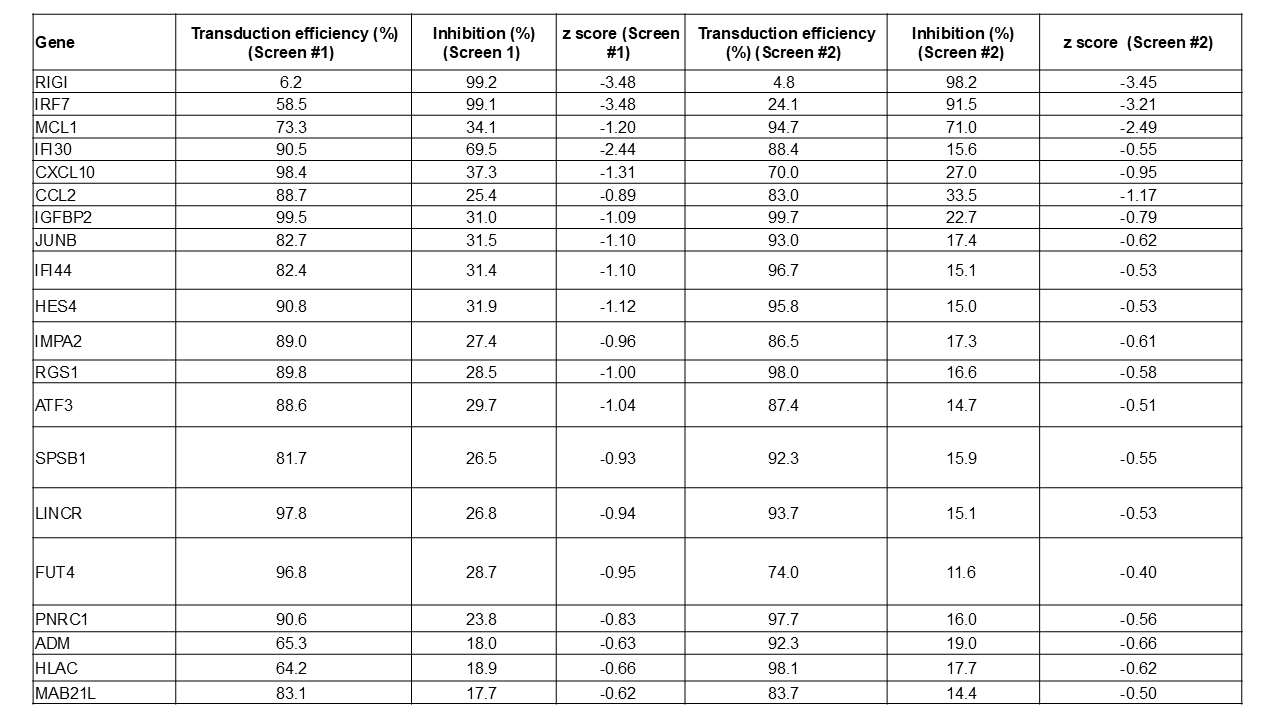

Supplement: Table S1 — ISG replicon screen results. [file jvi.00581-25-s0007.tif]
