# Supplementary material for: Interferon-stimulated gene MCL1 inhibits foot-and-mouth disease virus replication by modulating mitochondrial dynamics and autophagy
Source: J Virol. 2025 Jun 4;99(7):e00581-25. doi: 10.1128/jvi.00581-25 (PMC12282159; doi:10.1128/jvi.00581-25)
Supplement: Supplemental material — Supplemental methods and figure legends. [file jvi.00581-25-s0006.docx]

**Supplemental Materials and Methods**

*ISG Screen*

The lentivirus constructs used for the ISG screen was pTRIP.CMV.IVSb.ISG.ires.TagRFP^3^. To generate the pTRIP lentiviruses the plasmids pTRIP.CMV.IVSb.ISG.ires.TagRFP, pTRIP.CMV.IVSb.NLGP.ires.TagRFP, pTRIP.CMV.IVSb.VSV-G.ires.TagRFP were transfected in a ratio of 5 µg: 4 µg: 1 µg using XtremeGene9 (Sigma) transfection reagent. To screen interferon stimulating genes (ISGs) for antiviral activity, BHK-21 cells in 96-well plates (7 plates total per screen) were transduced with ISG lentiviruses expressing RFP (370 genes) for 48h. FMDV A24-GFP replicon RNA was transcribed in vitro using MEGAscript T7 kit (Ambion). To the transduced cells, 400ng per well of FMDV A24-GFP replicon RNA was transfected into the BHK cells for 6 hours using Lipofectamine 2000 (Invitrogen). At 6 hours post transfection, cells were fixed with 4% Paraformaldehyde, permeabilized with Triton X-100 (0.1%), and stained for GFP-positive cells and DAPI. All plates were imaged using ImageXPressMicro System (Molecular Devices) that captured 4 fields per well and data was analyzed for total (DAPI), transduced (RFP), and transfected (Alexa488) cells using MetaXPress software version 5.1.0.46. The screen was done in duplicate

*Knockdown of MCL1 with siRNA*

MGPK-αvβ6 cells were seeded in 24 well plates and treated with 10 nM of negative control siRNA or a pool of two siRNAs targeting MCL1 (IDT). After 24 hours, transfected cells were infected with A12 WT MOI: 0.5. At 6 hours post infection supernatants were collected to determine virus titers by plaque assay on BHK-21 cells.

*Cell Cycle Analysis of FMDV infected cells.*

MGPK-αvβ6 cells were seeded in 6 well plates at a density of 1 x 10^6^ cells per well and incubated overnight at 37 °C. The next day the cells were infected with A12 WT MOI: 0.1 for 4 hours before the cells were fixed with ethanol. The same protocol mentioned before for cell cycle analysis was followed to determine cell cycle distribution.

*Immunofluorescence Assay on Starved Cells*

MGPK-αvβ6 cells were seeded on 24 well plates on coverslips at the cell density of 8 x 10^4^ cells per well. The cells were incubated overnight at 37 °C and then either treated with PBS for 1 hour to induce starvation or were mock treated with DMEM. The cells were subjected to the same protocol as mentioned above for immunofluorescence assay to detect the formation of p62 and LC3 puncta.

**Supplemental Figures**

***Supplemental Table 1****. Results of ISG Screen against FMDV.* This table indicates the summary of two independent ISG screens. Transduction efficiency was calculated as the ratio of RFP positive cells to the total number of cells. Percent replication of the replicon was calculated by determining the number double positive GFP and RFP cells divided by the number of RFP cells. To determine percent inhibition the percent replications of the ISG transduced cells were normalized to the Fluc control transduced cells. Z score was determined by subtracting the percent replication from the average of the population of genes tested and this difference was divided by the standard deviation of the population.

***Supplemental Figure 1.*** *Knockdown of MCL1 by siRNA.* (A) Western blot analysis of lysates from MGPK- αvβ6 cells either treated with lipofectamine, siRNAs against MCL1 (siMCL1), or negative control siRNA (NC). The blot was probed with antibodies for MCL1 and actin. (B) The graph demonstrates the band intensity of the western blot for MCL1 which has been normalized to the actin loading control. (C) MGPK-αvβ6 were treated with either a negative control siRNA or siRNAs against MCL1 for 24 hours. The transfected cells were infected with A12 WT at an MOI of 0.5 for 6 hours (n=3). Virus titers were determined by plaque assay on BHK-21 cells. Statistical analysis was done by Student’s t-test.

***Supplemental Figure 2.*** *Cell cycle analysis of FMDV infected cells.* MGPK-αvβ6 cells were mock infected or infected with A12 WT MOI: 0.1 for four hours before being fixed and stained with DAPI (A). The graph represents the percent distribution of each cell cycle phase (G0/G1, S, G2/M) in mock infected or infected cells (B). Statistical analysis was done by Student’s t-test..

***Supplemental Figure 3.*** *Mito stress test on porcine cells*. (A) Mito Stress test was conducted with Seahorse bioanalyzer on MGPK- αvβ6 cells that were mock infected or infected with FMDV A12 WT MOI: 5. Subsequent addition of oligomycin, FCCP, and Rotenone/antimycin A allowed for calculation of different types of respiration occurring in mitochondria. (B) Basal respiration related OCR from Mito Stress test in (A). Basal respiration is oxygen consumption under basal conditions to meet the ATP demand of the cells and also due to proton leak. (C) ATP production OCR from Mito Stress test in (A). ATP production is oxygen consumption required to create ATP and is measured after addition of oligomycin. (C) Coupling efficiency from Mito Stress test from (A). Coupling efficiency refers to how efficient mitochondria are at consuming oxygen coupled to ATP production versus proton leak. It is calculated by dividing ATP linked OCR with basal respiration linked OCR. Statistical analysis was performed using one-way ANOVA with the Tukey *post hoc* test.

***Supplemental Figure 4.*** *Analysis of autophagic flux with p62 puncta formation in starved porcine cells.* (A) Indirect immunofluorescence imaging of p62 puncta in MGPK-αvβ6 cells that were starved for 1 hour, compared to cells that were not starved. The p62 protein was visualized using a polyclonal rabbit antibody and a secondary Alexa Fluor 488 antibody. The scale bar is representative for 10 µm. (B) Analysis of the number of p62 puncta was done with Fiji, and the graph represents the average number of puncta for each sample type (n=5). Statistical analysis was performed using Student’s t-test.

***Supplemental Figure 4.*** *Analysis of autophagic flux with p62 puncta formation in starved porcine cells.* (A) Indirect immunofluorescence imaging of p62 puncta in MGPK-αvβ6 cells that were starved for 1 hour, compared to cells that were not starved. The p62 protein was visualized using a polyclonal rabbit antibody and a secondary Alexa Fluor 488 antibody. The scale bar is representative for 10 µm. (B) Analysis of the number of p62 puncta was done with Fiji, and the graph represents the average number of puncta for each sample type (n=5). Statistical analysis was performed using Student’s t-test.

***Supplemental Figure 5.*** *Analysis of autophagic flux with LC3 puncta formation in starved porcine cells.* (A) Indirect immunofluorescence imaging of LC3 puncta in MGPK-αvβ6 cells that were starved for 1 hour, compared to cells that were not starved. The LC3 protein was visualized using a monoclonal rabbit antibody and a secondary Alexa Fluor 488 antibody. The scale bar is representative for 10 µm. (B) Analysis of the number of LC3 puncta was done with Fiji, and the graph represents the average number of puncta for each sample type (n=5). Statistical analysis was performed using Student’s t-test.
